# Supplementation with a selective amino acid formula ameliorates muscular dystrophy in *mdx* mice

**DOI:** 10.1038/s41598-018-32613-w

**Published:** 2018-10-02

**Authors:** Stefania Banfi, Giuseppe D’Antona, Chiara Ruocco, Mirella Meregalli, Marzia Belicchi, Pamela Bella, Silvia Erratico, Elisa Donato, Fabio Rossi, Francesco Bifari, Caterina Lonati, Stefano Campaner, Enzo Nisoli, Yvan Torrente

**Affiliations:** 1 Department of Pathophysiology and Transplantation, Università degli Studi di Milano, Fondazione IRCCS Ca’ Granda Ospedale Maggiore Policlinico, Centro Dino Ferrari, 20122 Milan, Italy; 2 Department of Public Health, Molecular and Forensic Medicine, and Sport Medicine Centre Voghera, University of Pavia, Pavia, 27100 Italy; 3 Center for Study and Research on Obesity, Department of Medical Biotechnology and Translational Medicine, University of Milan, Milan, 20129 Italy; 4 Novystem, Milan, 20129 Italy; 5 Centre for Genomic Science of IIT@SEMM, Istituto Italiano di Tecnologia, Milan, 20139 Italy; 6 Division of Stem Cells and Cancer, Deutsches Krebsforschungszentrum, Heidelberg, Germany; 7 Heidelberg Institute for Stem Cell Technology and Experimental Medicine, Heidelberg, Germany; 8 Laboratory of Cell Metabolism and Regenerative Medicine, Department of Medical Biotechnology and Translational Medicine, University of Milan, Milan, 20129 Milan, Italy; 9 Center for Surgical Research, Fondazione IRCCS Ca’ Granda, Ospedale Maggiore Policlinico, Milan, 20122 Italy

## Abstract

Duchenne muscular dystrophy (DMD) is one of the most common and severe forms of muscular dystrophy. Oxidative myofibre content, muscle vasculature architecture and exercise tolerance are impaired in DMD. Several studies have demonstrated that nutrient supplements ameliorate dystrophic features, thereby enhancing muscle performance. Here, we report that dietary supplementation with a specific branched-chain amino acid-enriched mixture (BCAAem) increased the abundance of oxidative muscle fibres associated with increased muscle endurance in dystrophic *mdx* mice. Amelioration of the fatigue index in BCAAem-treated *mdx* mice was caused by a cascade of events in the muscle tissue, which were promoted by endothelial nitric oxide synthase (eNOS) activation and vascular endothelial growth factor (VEGF) expression. VEGF induction led to recruitment of bone marrow (BM)-derived endothelial progenitors (EPs), which increased the capillary density of dystrophic skeletal muscle. Functionally, BCAAem mitigated the dystrophic phenotype of *mdx* mice without inducing dystrophin protein expression or replacing the dystrophin-associated glycoprotein (DAG) complex in the membrane, which is typically lost in DMD. BCAAem supplementation could be an effective adjuvant strategy in DMD treatment.

## Introduction

Duchenne muscular dystrophy (DMD) and Becker muscular dystrophy are caused by mutations within the *dystrophin* gene^1^ which primarily results in sarcolemmal fragility, muscle damage and respiratory or cardiac muscle fatigue and failure^2,3^. Treatment strategies for DMD have been done using genetic^4–9^, pharmacological^10,11^, or cellular^12–16^ approaches aimed at restoring dystrophin-associated glycoprotein (DAG) complex, reversing sarcolemmal fragility, and abating muscular dystrophy. However many hurdles remain and DMD is still incurable. Dysregulation of pathways associated with muscle fibre plasticity and angiogenesis in DMD are not well understood. Elucidation of such pathways may reveal signalling targets that are amenable to therapeutic manipulation by synthetic drugs. Activation of AMP-activated protein kinase (AMPK) ameliorates DMD mitochondrial activity and promotes oxidative slow-twitch myogenesis in *mdx* mice^17,18^. The transcription factor peroxisome proliferator-activated receptor γ coactivator-1α (PGC-1α) regulates the neuromuscular junction gene programme, induces a fast-to-slow fibre type transition, and ameliorates DMD pathology^19,20^. The dystrophin protein is expressed not only in skeletal muscle cells but also in vascular smooth muscle and endothelial cells (ECs)^21,22^. Vascular defects, including ultrastructural abnormalities of microvessels, mixed degenerating and regenerating capillaries, replication of the capillary basal lamina, and compression of capillaries and small-calibre veins by nodular proliferative connective tissue, have been described in DMD muscles^23–25^. Angiogenic factors, such as VEGF stimulate muscle regeneration in DMD^26–28^. Based on the benefits of pro-oxidative and angiogenic regulation in muscular dystrophy, we and other groups introduced the concept of dietary supplementation to ameliorate dystrophic muscle pathology^29,30^. Creatine, taurine, l-glutamine, and l-arginine have been shown to have limited benefits on muscle strength and dystrophic features, though these supplements remain to be formally evaluated in long-term studies^31–34^.

Angiogenesis in skeletal muscle is regulated by nitric oxide (NO) produced by endothelial nitric oxide synthase (eNOS), neuronal NOS (nNOS)^35,36^, inducible NOS (iNOS). All of these isoforms are expressed in muscle, and nNOS, in particular, is abundant at the surfaces of type II (fast-twitch) fibres, and less so in type I (slow-twitch) fibres, bound to the sarcolemma via the dystrophin-glycoprotein complex. On the other hand, eNOS is expressed on the cell surfaces of ECs in a complex with caveolin-1 and dystrophin and in type I fibres mitochondrial membrane level^37,38^. In both cases, several reports have shown that oestrogens, *via* oestrogen-related receptor-α (ER-α), can upregulate eNOS mRNA and protein levels, increasing the phosphorylation of eNOS via specific kinases^39^. Dystrophin deficiency induces a selective defect in flow-dependent mechanotransduction, thus attenuating flow (shear stress)-mediated endothelium-dependent dilation (FMD) and eNOS expression, and may contribute to low arteriolar density^40^. Furthermore, eNOS deletion in skeletal muscle impairs muscle performance^41^. We have demonstrated that eNOS is involved in the effect of dietary supplementation with a selective branched-chain amino acid-enriched mixture (BCAAem) on PGC-1α and sirtuin 1 expression in cardiac and skeletal muscles of middle-aged mice^42^. Here, we describe for the first time that BCAAem promotes muscle repair by increasing oxidative fibre content and angiogenic remodelling in dystrophic skeletal muscle in *mdx* mice. We found a prevalence of myosin heavy chain (MyHC) type IIx aerobic/glycolytic and MyHC type IIb glycolytic muscle fibres in the BCAAem-treated *mdx* muscles associated with increased response to fatigue. We demonstrate that the molecular mechanism underlying BCAAem action is based on augmented eNOS expression and phosphorylation and with increased synthesis of VEGF and recruitment of bone-marrow-derived Sca1^+^CD34^+^ endothelial progenitors to dystrophic muscles. This BCAAem response determines the microvasculature reorganization of dystrophic *mdx* skeletal muscle. The pivotal role of eNOS in mediating the effects of BCAAem on EC compartments was demonstrated by the absence of a BCAAem response in eNOS null mutant mice (*eNOS*^−/−^). The increase in oxidative muscle fibre content and the angiogenic remodelling mediated by BCAAem mitigate muscle damage in *mdx* mice, supporting the notion that dietary supplementation with BCAAem is an effective adjuvant strategy for DMD treatment.

## Results

### BCAAem induces an increase in oxidative fibre content in skeletal muscles of *mdx* mice

**Figure 1:**
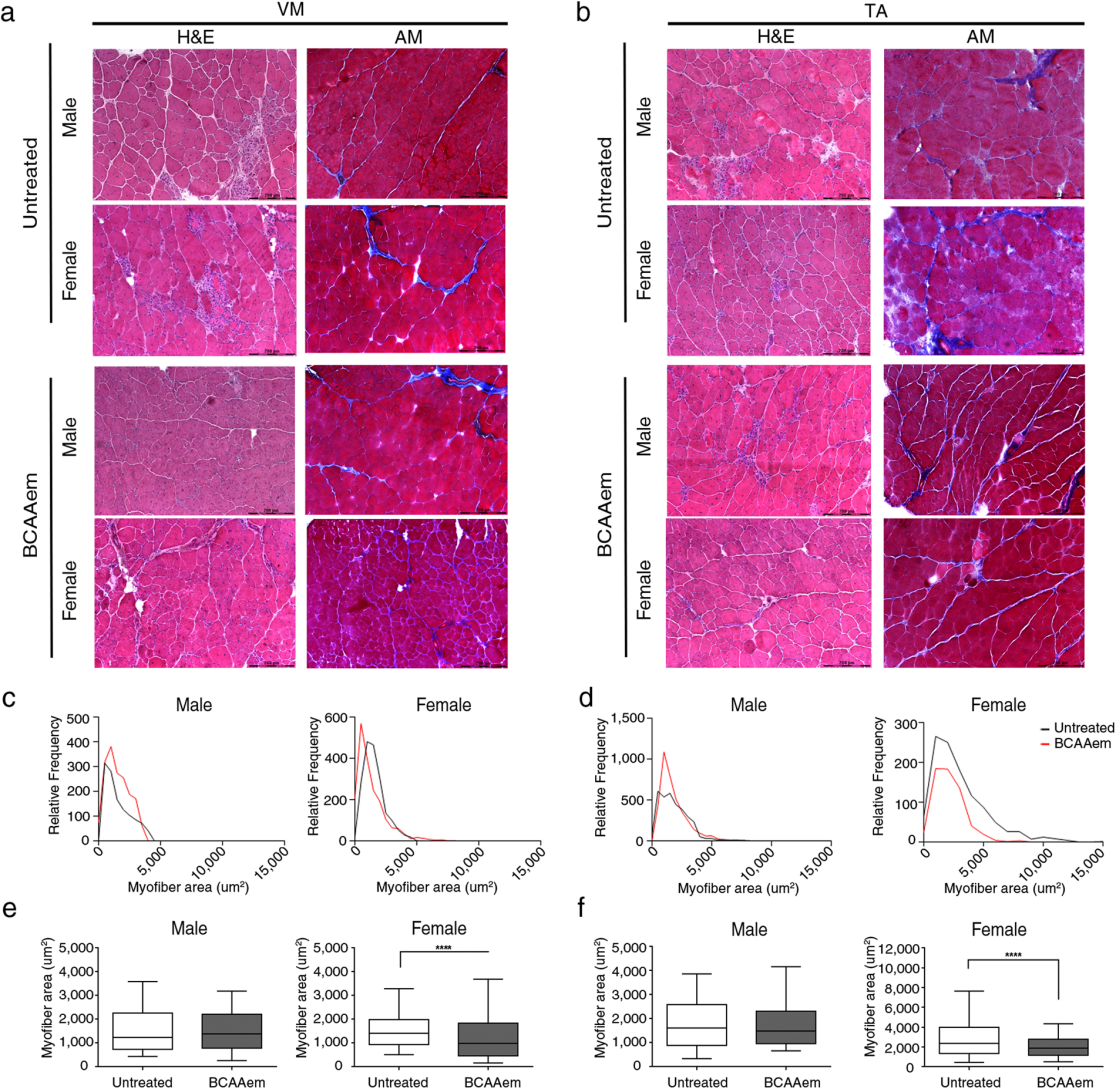
Haematoxylin and eosin (H&E) and Azan-Mallory (AM) analysis of BCAAem-treated and untreated *mdx* male and female mice. Representative H&E and AM staining of *vastus medialis* (VM) (**a**) and *tibialis anterior* (TA) (**b**) of untreated and BCAAem-treated *mdx* male and female mice. Scale bars, 200 μm. Quantification of the relative frequency of the myofibre cross-sectional area (CSA) expressed as the frequency distribution of the VM (**c**,**e**) and TA (**d**,**f**) muscles of the untreated and treated experimental groups of male and female mice. Boxes indicate 25^th^ to 75^th^ percentiles; whiskers indicate 5^th^ to 95^th^ percentiles and the line indicates the median (n = 5 per group). Statistical error analysis was performed by two-way ANOVA with Bonferroni correction; ****p < 0.0001 for the indicated comparisons.

To study the effects of BCAAem supplementation on muscular dystrophy, we administered the amino acid mixture (1.5 mg/g body weight) with drinking water to 3-month-old *mdx* mice for two weeks. This genetically dystrophic mouse model exhibits dystrophic muscle features and skeletal muscle vascular regression^22,40,43,44^. Since a greater level of muscular damage and fibrosis have been found in ageing female *mdx* mice than in males^45,46^, all groups of animals were equally divided into males and females and separately analysed. BCAAem-treated male (n = 5) and female (n = 5) *mdx* mice were compared to age-matched untreated male (n = 5) and female (n = 5) *mdx* mice and to BCAAem-treated and untreated wild-type *C57BL6/J* and *eNOS*^−/−^ mice (5 males and 5 females in each group) (Figs 1 and S2). *eNOS*^−/−^ mice were included in this study because we previously reported that BCAAem treatment increased eNOS expression and activation (eNOS-Ser1177 phosphorylation) in cardiomyocytes, whereas eNOS KO cells were insensitive to BCAAem^42^.

We measured the circulating BCAA (leucine, isoleucine, and valine) levels in *mdx*, wild-type (*C57BL6/J*) and *eNOS*^−/−^ mice acutely (30–120 min) supplemented with BCAAem as previously described^42^. The BCAA levels in the plasma of *C57BL6/J* and *mdx* mice had superimposable kinetics (Fig. S1). Moreover, we have demonstrated that water consumption was the same by all untreated and BCAAem-treated mice (Fig. S1).

**Figure 2:**
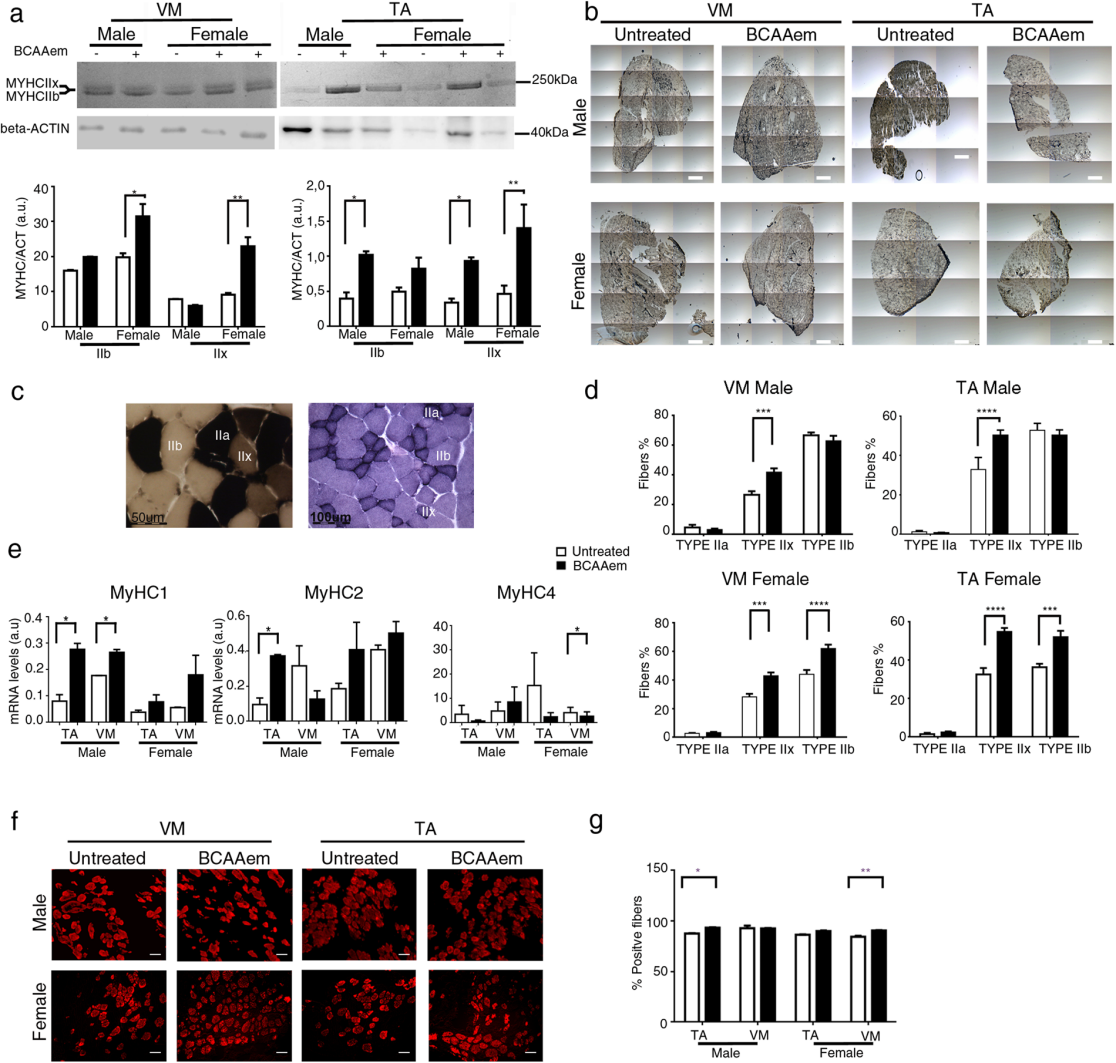
Characterization of the MyHC isoforms in the muscles of BCAAem-treated and untreated *mdx* mice. Cropped image of a representative Coomassie-Blue-stained protein gel showing electrophoretic separation of the MyHC IIx and IIb isoforms from the total protein lysates of VM and TA muscles of male and female *mdx* mice. (**a**) Cropped image of a western blot of β-actin from the total protein lysates of VM and TA muscles of male and female *mdx* mice. (**a**) Full-length Coomassie-stained gels and blots are presented in Supplementary Fig. S8. Quantification of the myosin/actin ratio to describe the different amounts of myosin isoforms. (**a**) Whole field (**b**) (scale bar, 500 µm) and higher magnification (**c**) (scale bar, 50 µm) of ATPase (pH 4.3) (left) and SDH-stained (right, scale bar, 100 µm) sections for detection of the distribution and composition of myosin heavy chain (MyHC) isoforms (**d**) of BCAAem-treated and untreated *mdx* groups. qRT-PCR quantification analysis of expression of the MYH1, MYH2 and MYH4 genes in the VM and TA muscles of BCAAem-treated and untreated male and female *mdx* mice (n = 5 per group) normalized to expression of the GAPDH housekeeping gene. (**e**) Fast MyHC isoform immunofluorescence staining of BCAAem-treated and untreated VM and TA *mdx* male and female muscle sections (**f**) (scale bar 100 µm). The fast MyHC isoform fluorescence was quantified with the ImageJ software 6.0 and the corresponding histograms are reported as percentage of positive fibers per section. (**g**) All the experiments were conducted in triplicate. All data are presented as the mean ± s.e.m. Statistical error analysis was performed by two-way ANOVA with Bonferroni correction; *p < 0.05, **p < 0.01, ***p < 0.001, and ****p < 0.0001 indicate comparisons that reflect significant differences relative to the untreated group.

Morphometric analysis of the *tibialis anterior* (TA) and *vastus medialis* (VM) muscles of BCAAem-treated *mdx* mice did not show reduction in the levels of the typical fibrotic infiltrate and centrally nucleated fibres (Fig. 1a, b). *Mdx* mice were characterized by a high variability in myofibre size (Fig. 1c, d). The cross-sectional areas (CSAs) of the myofibres observed in the muscles of BCAAem-treated *mdx* females (TA: 2792 ± 53 μm^2^, n = 5; VM: 1329 ± 27 μm^2^, n = 5) were significantly lower than those observed in the muscles of untreated mice (TA: 4519 ± 98 μm^2^, n = 5; VM: 1564 ± 20 μm^2^, n = 5) (p < 0.0001, one-way analysis of variance (ANOVA) with Bonferroni correction). Although not significant, similar reduction in CSAs was observed in the muscles of BCAAem-treated *mdx* males (TA: 2945 ± 44.79 μm^2^, n = 5; VM: 1538 ± 22 μm^2^, n = 5) compared to the CSAs observed in the muscles of untreated* mdx* males (TA: 4206 ± 88.4 μm^2^, n = 5; VM: 1571 ± 30 μm^2^, n = 5) (Fig. 1e, f). Moreover, the coefficient of variation in myofibre area was significantly higher for the muscles of BCAAem-treated *mdx* male and female mice (TA: 67.14 ± 5.35% for males and 48 ± 3.12% for females; VM: 69 ± 4.75% for males and 52 ± 4.23% for females) than for those of untreated *mdx* male and female mice (TA: 51.32 ± 7% for males and 50.73 ± 2.5% for females; VM: 40 ± 3.88% for males and 52 ± 4.23% for females) (p < 0.0001, F-test of variance). Histological, biochemical, and morphometric analyses of BCAAem-treated and untreated *eNOS*^−/−^ mice revealed unmodified muscle phenotypes (Fig. S2). In particular, the areas of the muscle fibres of BCAAem-treated *eNOS*^−/−^ mice (TA: 2103 ± 42 μm^2^ for males and 2159 ± 55 μm^2^ for females; VM: 2374 ± 45 μm^2^ for males and 2111 ± 39 μm^2^ for females) were similar to those observed in untreated mice (TA: 1905 ± 46 μm^2^ for males and 1886 ± 32 μm^2^ for females; VM: 2489 ± 63 μm^2^ for males and 2075 ± 41 μm^2^ for females) (Fig. S2g–j). However, a statistically significant reduction in CSA values was also observed for the TA (1813 ± 19 μm^2^, n = 5) and VM (2476 ± 27 μm^2^) muscles of BCAAem-treated male *C57BL6/J* mice compared to the CSAs of untreated TA (1871 ± 22.5 μm^2^) and VM (2679 ± 55 μm^2^) muscles of *C57BL6/J* males, with a similar trend observed for females (TA: untreated 2951 ± 68 μm^2^ vs. treated 2110 ± 51 μm^2^; VM: untreated 2479 ± 50 μm^2^ vs. treated 2687 ± 48 μm^2^) (p < 0.001, one-way ANOVA with Bonferroni correction) (Fig. S2k, l). Changes in the CSAs of the muscles of dystrophic *mdx* and wild-type *C57BL6/J* mice suggest the existence of a muscle remodelling process induced by BCAAem that could be associated with a change in muscle fibre content. Because the myosin motor protein is closely associated with sex-based differences in muscle function, we verified the fibre composition of both sexes in untreated and BCAAem-treated mice. We observed a statistically significant increase in the expression of the MyHC IIx (oxidative/glycolytic type IIx myofibres) protein in the TAs and VMs of BCAAem-treated mdx females and the TAs of *mdx* males, whereas MyHC IIb (glycolytic type IIb myofibres) expression was significantly increased in the TAs of BCAAem-treated *mdx* males and VMs of BCAAem-treated *mdx* females (p < 0.05, one-way ANOVA with Bonferroni correction) (Fig. 2a). To confirm these findings, myofibrillar adenosine triphosphatase (mATPase) activity was evaluated at pH 4.3 by using an enzymatic stain that distinguishes between slow and fast glycolytic myofibres (Fig. 2b–d). Similar to the ATPase assay, Succinate Dehydrogenase (SDH) activity was tested to verify the distribution of fast and slow myofibers (Fig. 2c). ATPase staining confirmed the statistically significant increase in MyHC IIx levels (p < 0.001, two-way ANOVA with Bonferroni correction) in males and in the levels of both MyHC IIx and MyHC IIb myofibres (p < 0.001, two-way ANOVA with Bonferroni correction) in females of the BCAAem-treated group compared with the untreated *mdx* mice (Fig. 2b–d). All these results were confirmed by RT-PCR analysis of the MYH 1, 2, and 4 genes (Fig. 2e). To elaborate, the levels of MYH1, which encodes the MyHC IIx isoform, were significantly increased in the TA and VM muscles of BCAAem-treated *mdx* mice of both sexes. Immunofluorescence analysis was also performed to measure the proportions of oxidative/slow and glycolytic/fast myofibres in the TA and VM muscles of male and female *mdx* mice. The number of slow MyHC isoforms was statistically significant higher (p < 0.0001, two-way ANOVA with Bonferroni correction) in the TAs and VMs of BCAAem-treated mice than in untreated female *mdx* mice, whereas significant increase was observed in the TAs of BCAAem-treated male *mdx* mice (p < 0.05, two-way ANOVA with Bonferroni correction) (Fig. S3). Conversely, the number of fast/glycolytic myofibres was significantly increased in the TAs of male and VMs of female BCAAem-treated *mdx* mice than untreated mice (Fig. 2f, g; p < 0.05 and p < 0.01 respectively, two-way ANOVA with Bonferroni correction). Taken together, these results suggest that BCAAem supplementation promotes an increase in the oxidative/glycolytic type IIx and glycolytic MyHC IIb content of *mdx* males and females.

### BCAAem ameliorates the motor function of *mdx* mice

**Figure 3:**
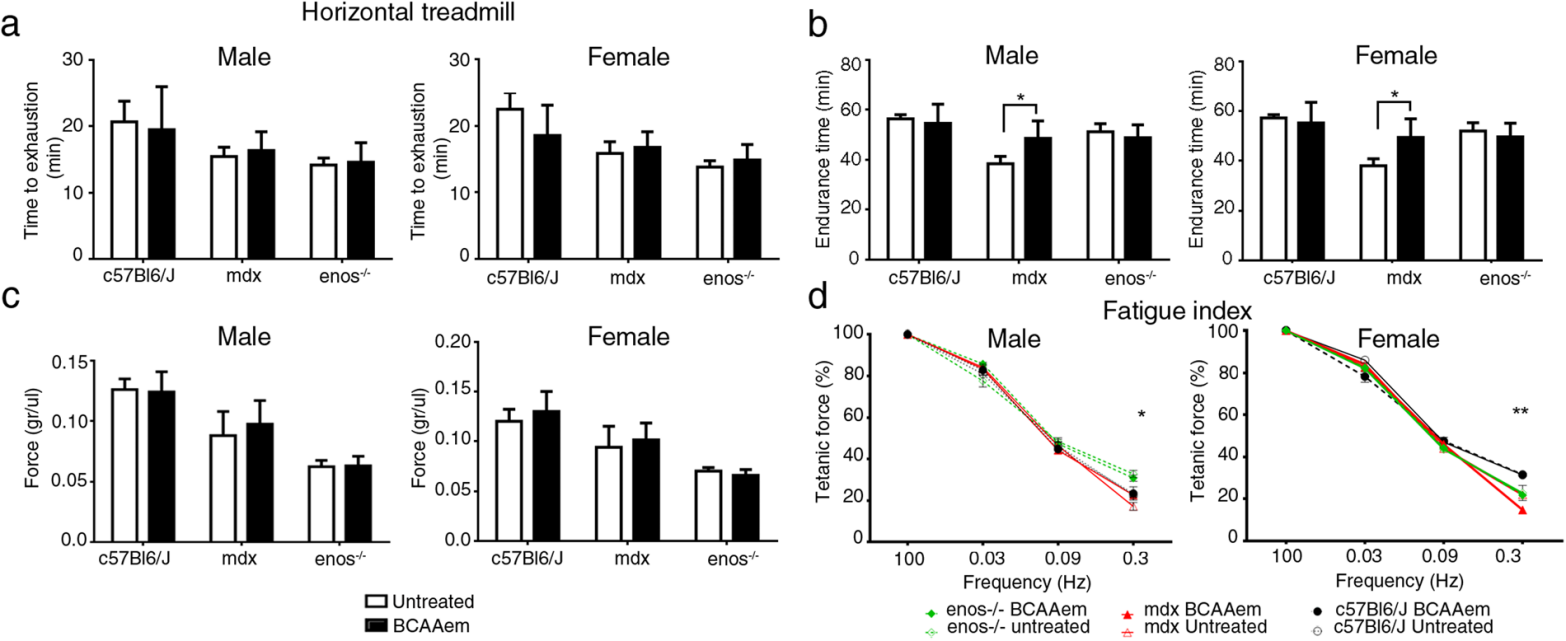
BCAAem ameliorates fatigue resistance in *mdx* mice. *In vivo* muscle function was determined by an incremental treadmill exhaustion test. Exhaustion time is expressed in min (n = 10; five females and five males for each genotype). (**a**) *In vivo* endurance time is expressed in min. (**b**) Muscle function was evaluated *in vitro* by measuring the tetanic force (g/µL) of single fibres isolated from TA strips. (**c**) Fatigue index, defined as the decrease in tetanic force at different stimulation frequencies (from 0.03 to 0.3 Hz), is compared to the maximal tetanic force (Tf) and expressed as a percentage. Frequency 0.3 Hz; *p < 0.05 for untreated vs. BCAAem-treated *mdx* male mice; **p < 0.01 for untreated vs. BCAAem-treated *mdx* female mice. (**d**) All data are presented as the mean ± s.e.m. Statistical error analysis was performed by two-way ANOVA with Bonferroni correction.

Then, we verified whether the increased proportion of myofibres expressing type IIx and IIb myosin, promoted by the dietary BCAAem supplementation, led to sustained recovery of muscle function in muscular dystrophy. Overall, motor capacity was assessed in terms of time to exhaustion and endurance time with treadmill performance^47^ in three-month-old *C57BL6/J* (n = 5; 3 females and 2 males)*, mdx* (n = 5; 3 females and 2 males), and *eNOS*^−/−^ (n = 5; 3 females and 2 males) mice. There was no clear amelioration in time to exhaustion after BCAAem administration in any of the analysed genotypes (Fig. 3a). Notably, the endurance time was ameliorated in BCAAem-treated *mdx* mice, with an increase of ~20% compared to the endurance time of untreated *mdx* animals (38.40 ± 2.98 min vs. 48.60 ± 3.12 min in untreated and treated males, respectively; 38 ± 2.76 min vs. 49.40 ± 3.33 min in untreated and treated females, respectively; mean ± s.e.m.; p < 0.05, two-way ANOVA with Bonferroni correction) (Fig. 3b). Conversely, amino acid supplementation was unable to increase the exhaustion time and endurance of both *C57BL6/J* and *eNOS*^−/−^ mice (Fig. 3a). Moreover, the normalized tetanic force and fatigue index were measured in isolated TA muscles obtained from mice of all genotypes. *Mdx* and *eNOS*^−/−^ mice showed lower basal values of TA-specific force than *C57BL6/J* mice (Fig. 3c) and did not show any significant amelioration after BCAAem treatment. Moreover, *C57BL6/J* mice did not show any improvement in TA-specific force after BCAAem treatment. Amelioration of tetanic force was evident at high stimulation frequency in both female (p < 0.01, two-way ANOVA with Bonferroni correction) and male (p < 0.05, two-way ANOVA with Bonferroni correction) BCAAem-treated *mdx* mice (Fig. 3d). BCAAem supplementation was unable to improve motor function in *eNOS*^−/−^ mice, suggesting that eNOS may be associated with the beneficial effects of amino acid supplementation (Fig. 3a–d). These data suggest that the difference in MyHC content observed between BCAAem-treated *mdx* males and females is not the only explanation for the motor function amelioration observed in BCAAem-treated *mdx* mice.

### BCAAem modulates the expression of eNOS and VEGF in dystrophic skeletal muscle of *mdx* mice

**Figure 4:**
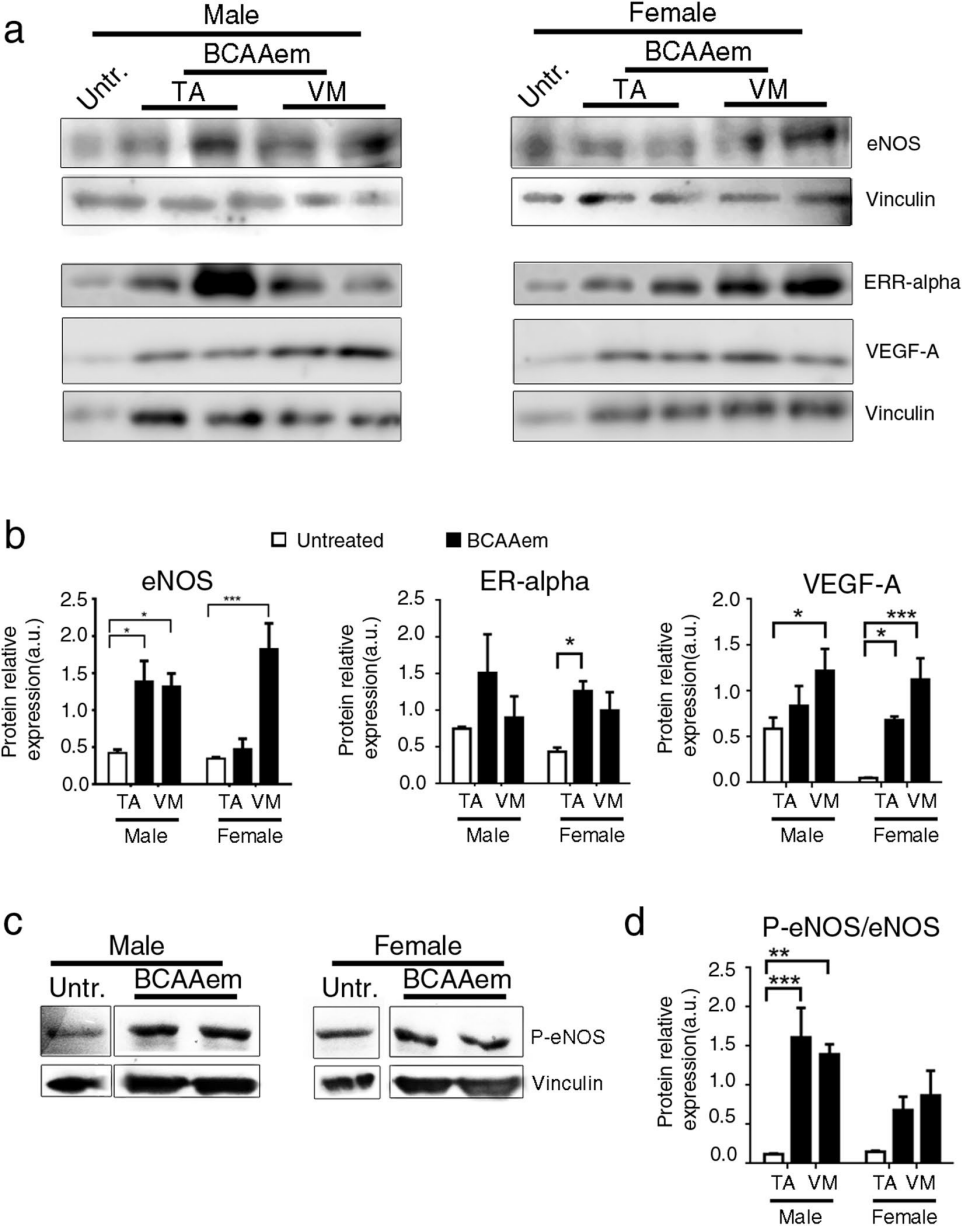
BCAAem administration increases eNOS, ERα and VEGF levels in dystrophic muscle tissue of *mdx* mice. Cropped image of a representative western blot showing the expression of the ERα, eNOS, VEGF and vinculin proteins in VM and TA muscle tissues of and untreated male and female *mdx* mice (representative VM and TA samples of two BCAAem-treated males and two BCAAem-treated females and one representative VM sample of untreated mice of each sex are shown). Full-length blots are presented in Supplementary Fig. S9. (**a**) Densitometric analyses are shown as the ERα/vinculin ratio, eNOS/vinculin ratio and VEGF/vinculin ratio. (**b**) Cropped image of a representative western blot (**c**) and densitometric analysis (**d**) of p-eNOS and vinculin proteins VM and TA muscle tissues of BCAAem-treated and untreated in male and female *mdx* mice. Full-length blots are presented in Supplementary Fig. S10. VM muscle of an untreated *mdx* mouse was used as a control. Data are shown as p-eNOS/vinculin ratios. All these experiments were performed three times. All data are presented as the mean ± s.e.m. Statistical error analysis was performed by two-way ANOVA with Bonferroni correction; *p < 0.05, **p < 0.01 and ***p < 0.001 indicate comparisons that reflect significant differences relative to the untreated group.

Since skeletal muscle is an oestrogen-responsive tissue with high expression of oestrogen receptor α (ERα)^48–50^ and to explain the prevalence in females of myofibres expressing type IIx and IIb myosins, which are associated with ameliorated endurance time in BCAAem-treated *mdx* mice, we compared the ERα expression levels in BCAAem-treated *mdx* males and females^51–53^. ERα protein expression was significantly higher in the TA muscles (p < 0.05, two-way ANOVA with Bonferroni correction) and higher in the VM muscles of BCAAem-treated mice than in untreated female *mdx* mice (Fig. 4a ,b). ERα protein expression was higher but not significantly different in the TA (p = 0.35, two-way ANOVA with Bonferroni correction) and VM (p = 0.72, two-way ANOVA with Bonferroni correction) muscles of BCAAem-treated mice than in untreated male *mdx* mice. The stimulatory effect of oestrogens and ERα on the release of several endothelial mediators, especially NO, has been well described^54^. Thus, we sought to investigate the effect of BCAAem supplementation on eNOS activation and the downstream signalling pathway. We performed a series of immunoblot assays on BCAAem-treated and untreated *mdx* mice. In response to amino acid supplementation, a statistically significant increase in eNOS expression was evident in the VM muscles of both male (p < 0.05, two-way ANOVA with Bonferroni correction) and female (p < 0.001, two-way ANOVA with Bonferroni correction) *mdx* mice and in the TA muscles (p < 0.05 two-way ANOVA with Bonferroni correction) (Fig. 4a, b) of male* mdx* mice. These responses were associated with increased eNOS phosphorylation (1170Ser-p-eNOS) in both the TA and VM muscles of *mdx* males and females (Fig. 4c, d; statistically significant for only the TA and VM muscles of mdx males, with p < 0.001 and p < 0.01, respectively; two-way ANOVA with Bonferroni correction). Thus, we measured VEGF-A protein expression as a downstream signalling effect of eNOS-dependent NO production^55^. BCAAem supplementation was able to increase VEGF-A expression in the muscles of male *mdx* mice, with statistical significance observed in the VM muscles (*p* < 0.05, two-way ANOVA with Bonferroni correction) (Fig. 4a, b). VEGF-A levels were significantly higher in the TA (*p* < 0.05, two-way ANOVA with Bonferroni correction) and VM (p < 0.001; two-way ANOVA with Bonferroni correction) muscles of BCAAem-treated *mdx* mice than in untreated females (Fig. 4a, b). All these data suggest that BCAAem promotes eNOS and VEGF expression in the skeletal muscles of *mdx* mice.

### BCAAem treatment ameliorates vascular rarefaction in the muscles of *mdx* mice

**Figure 5:**
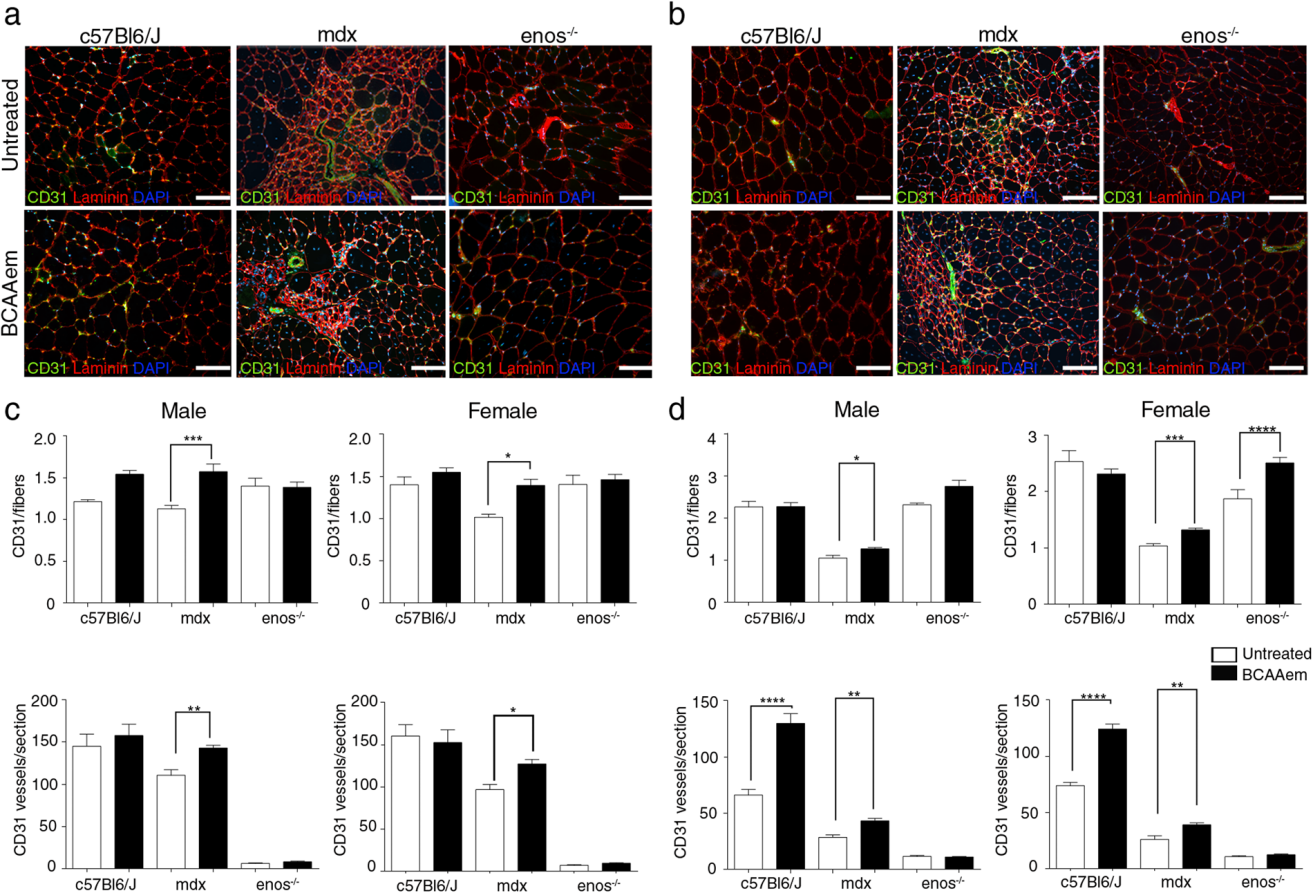
BCAAem treatment increases the number of CD31+ vessels in the muscles of *mdx* mice. Cryosections of VM (**a**) and TA (**b**) muscle tissues of BCAAem-treated and untreated male and female *C57BL6/J*, *mdx* and *eNOS*^−/−^ mice were analysed by immunofluorescence staining for CD31 and laminin (5 males and 5 females for each experimental group). Nuclei were stained with DAPI. Analysis of the VM and TA muscles of BCAAem-treated male and female *mdx* mice demonstrates an increase in the number of CD31+ vessels per fibre and per section. Scale bar, 100 µm. Quantification of CD31+ vessels per fibre and per section of VM (**c**) and TA (**d**) is shown. All data are presented as the mean ± s.e.m. Statistical error analysis was performed by one-way ANOVA with Bonferroni correction; *p < 0.05, **p < 0.01, ***p < 0.001, and ****p < 0.0001 indicate significant differences from the untreated group.

**Figure 6:**
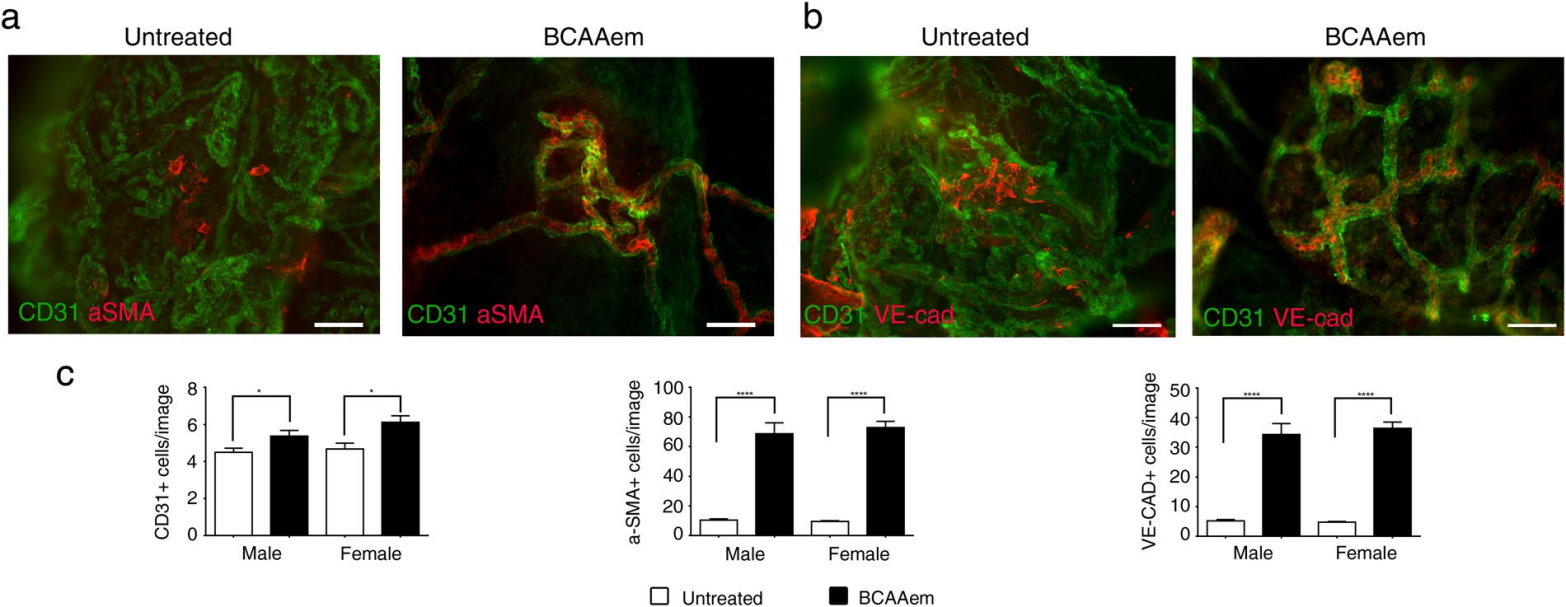
BCAAem treatment leads to amelioration of vessel architecture in muscles of *mdx* mice. Probe-based confocal laser endomicroscopy was performed in TAs of BCAAem-treated and untreated male and female *mdx* mice. Treated and untreated *mdx* mice were injected with CD31 Alexa-Fluor 488 and α-SMA Alexa-Fluor 647 (n = 5 per group) (**a**) and with CD31 Alexa-Fluor 488 and VE-cadherin Alexa-Fluor 647 (n = 5 per group) (**b**). Representative images show increased α-SMA+ pericyte vessel coverage and aligned VE-cad+ cells organized in regular wall structures. Scale bar, 200 µm. Quantification of the number of CD31+ vessels and α-SMA+ and VE-cad+ cells per field of view of the muscles of BCAAem-treated and untreated male and female mice is shown. (**c**) All data are presented as the mean ± s.e.m. Statistical error analysis was performed by two-way ANOVA with Bonferroni correction; *p < 0.05, ****p < 0.0001 indicate comparisons that reflect significant differences relative to the untreated group.

Thus, we examined the effect of BCAAem supplementation on blood vessels in the skeletal muscles of *mdx* (n = 10), *C57BL6/J* (n = 10), and *eNOS*^−/−^ (n = 10) mice. Each experimental group was composed of 5 male and 5 female mice. We measured vascular density by CD31 staining as a marker of capillary networks, followed by quantification of both total CD31^+^ blood vessels and CD31^+^ vessel-to-fibre ratio. As previously demonstrated^22,40,43^, the CD31^+^ vessel-to-fibre ratio and the CD31^+^ vessels/section ratio were lower in the muscles of the *mdx* mice than in the muscles of the control *C57BL6/J* mice (the decrease was ~56% in the TAs and ~18% in the VMs of both male and female animals) (Fig. 5). Notably, the CD31^+^ vessels/section ratio was substantially lower in both the TA and VM muscles of *eNOS*^−/−^ mice than in *C57BL6/J* mice, although the CD31^+^ vessel-to-fibre ratio was not different in the muscles of these two genotypes (Fig. 5). BCAAem supplementation increased the CD31^+^ vessel-to-fibre ratio in both the TA and VM muscles of male and female *mdx* mice (Fig. 5). Except for an increase in the CD31+ vessels/section ratio in the VM (not statistically significant) and TA (p < 0.001, one-way ANOVA with Bonferroni correction) muscles of BCAAem-treated compared to the values observed for untreated *C57BL6/J* mice, no differences were observed in either type of muscle in *eNOS*^−/−^ mice of either sex with or without amino acid treatment (Fig. 5). These results suggest a pivotal role for eNOS in mediating the effect of BCAAem on endothelial compartments. To analyse the effect of BCAAem on the vessel structures of dystrophic skeletal muscles, we performed *in vivo* probe-based confocal laser endomicroscopy (p-CLE) to examine the expression of CD31 and the mural cell marker α-SMA. This analysis showed that few pericytes covered blood vessels in the TA muscles of both male and female *mdx* mice, thus indicating immature vessel architecture (Fig. 6a). In BCAAem-treated *mdx* mice, the vascular architecture of TA muscles was less chaotic and complex, exhibiting improved vessel coverage by pericytes. This result the existence of mature vessels, as confirmed by an increase in the number of α-SMA+ cells (Fig. 6c). We also analysed vessel morphology in the TA muscles of *mdx* mice by p-CLE analysis of VE-cadherin and CD31 expression (Fig. 6b). The TAs of untreated *mdx* mice showed irregular vessel walls, whereas the TA muscles of BCAAem-treated male *mdx* mice showed highly uniform alignment of the VE-cadherin+ vessels (Fig. 6b). Quantification of fluorescence signals showed that the CD31+, α-SMA+, and VE-cadherin+ cell numbers were statistically higher in the skeletal muscles of BCAAem-treated mice of both sexes (Fig. 6c). These data indicate that amino acid supplementation can improve vessel structure in the skeletal muscles of BCAAem-treated dystrophic males and females.

### BCAAem ameliorates the vascularization of dystrophic muscles via bone-marrow-derived endothelial progenitors

**Figure 7:**
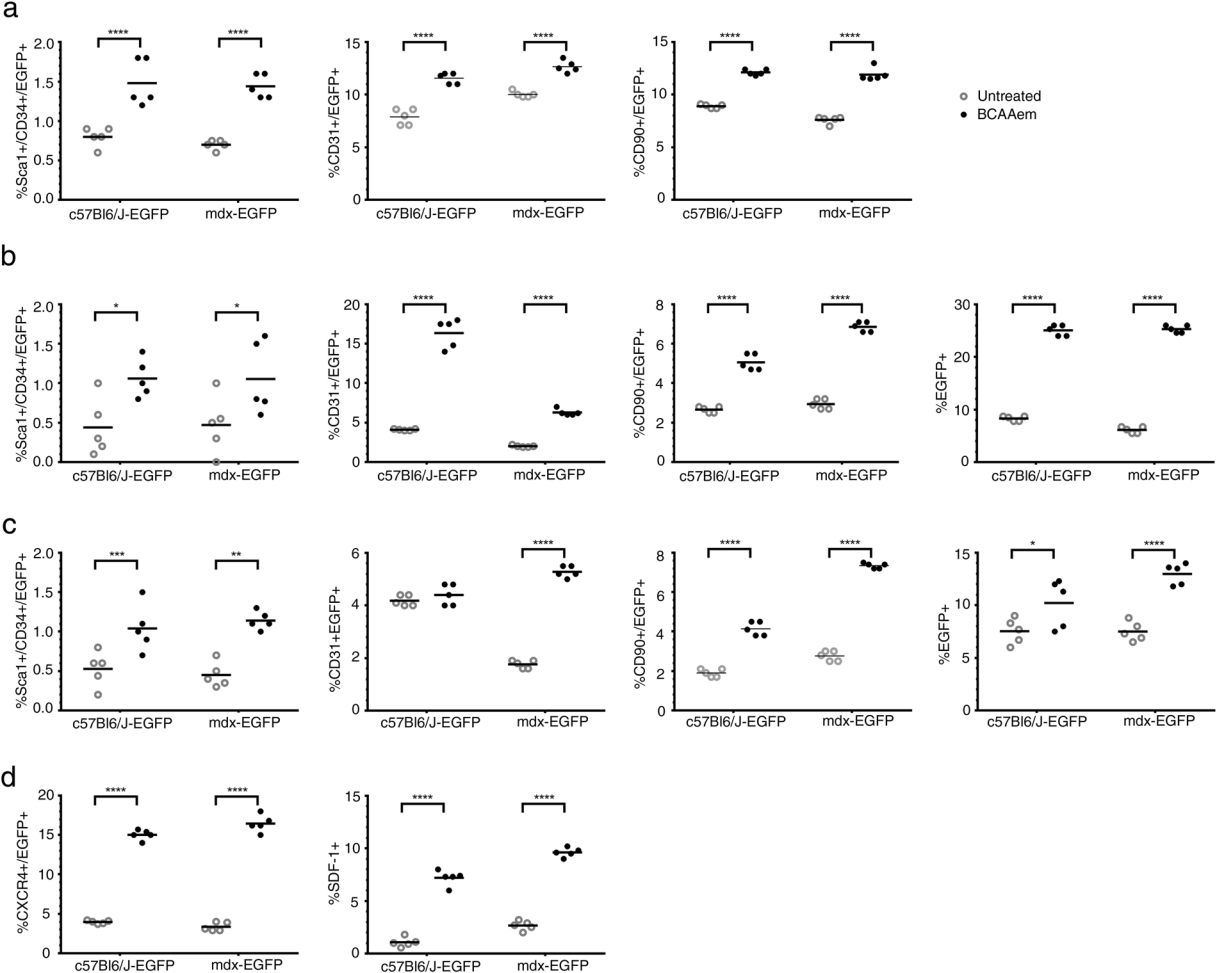
BCAAem promotes the mobilization of donor bone marrow-derived EGFP endothelial progenitors to dystrophic muscles. Bone marrow (BM) of donor *C57BL6/J-EGFP* mice was transplanted into lethally irradiated host *C57BL6/J* and *mdx* mice (referred to after transplantation as *C57BL6/J-EGFP* and *mdx-EGFP* mice). BM-transplanted animals were treated with BCAAem and compared to untreated animals. Flow cytometric analysis of SCA1+ CD34+ EGFP+ EPs and CD31+/CD90+ EGFP+ endothelial cells (ECs) was performed in blood (**a**) and in TA (**b**) and VM (**c**) muscle tissue isolated from BCAAem-treated and untreated *C57BL6/J-EGFP* and *mdx-EGFP* mice (n = 5 for each experimental group). FACS analysis of CXCR4+ EGFP+ and SDF-1+ cells (**d**) is shown in TA muscle of *C57BL6/J-*EGFP and *mdx-EGFP* mice. Quantification of EGFP+ cells is shown as percentage of total isolated cells for TA and VM. The increase in the number of EPs in the muscles of BCAAem-treated *mdx-EGFP* mice demonstrates that BCAAem supplementation stimulates muscle homing of BM stem cells. The muscle homing effect of BCAAem is supported by the increase in the levels of CXCR4+/EGFP+ and SDF-1+ cells (n = 5 for each experimental group). Individual data are shown in the graphs, and the line indicates the mean value. Statistical error analysis was performed by two-way ANOVA with Bonferroni correction; *p < 0.05, **p < 0.01, and ****p < 0.0001 indicate comparisons that reflect significant differences relative to the untreated group.

**Figure 8:**
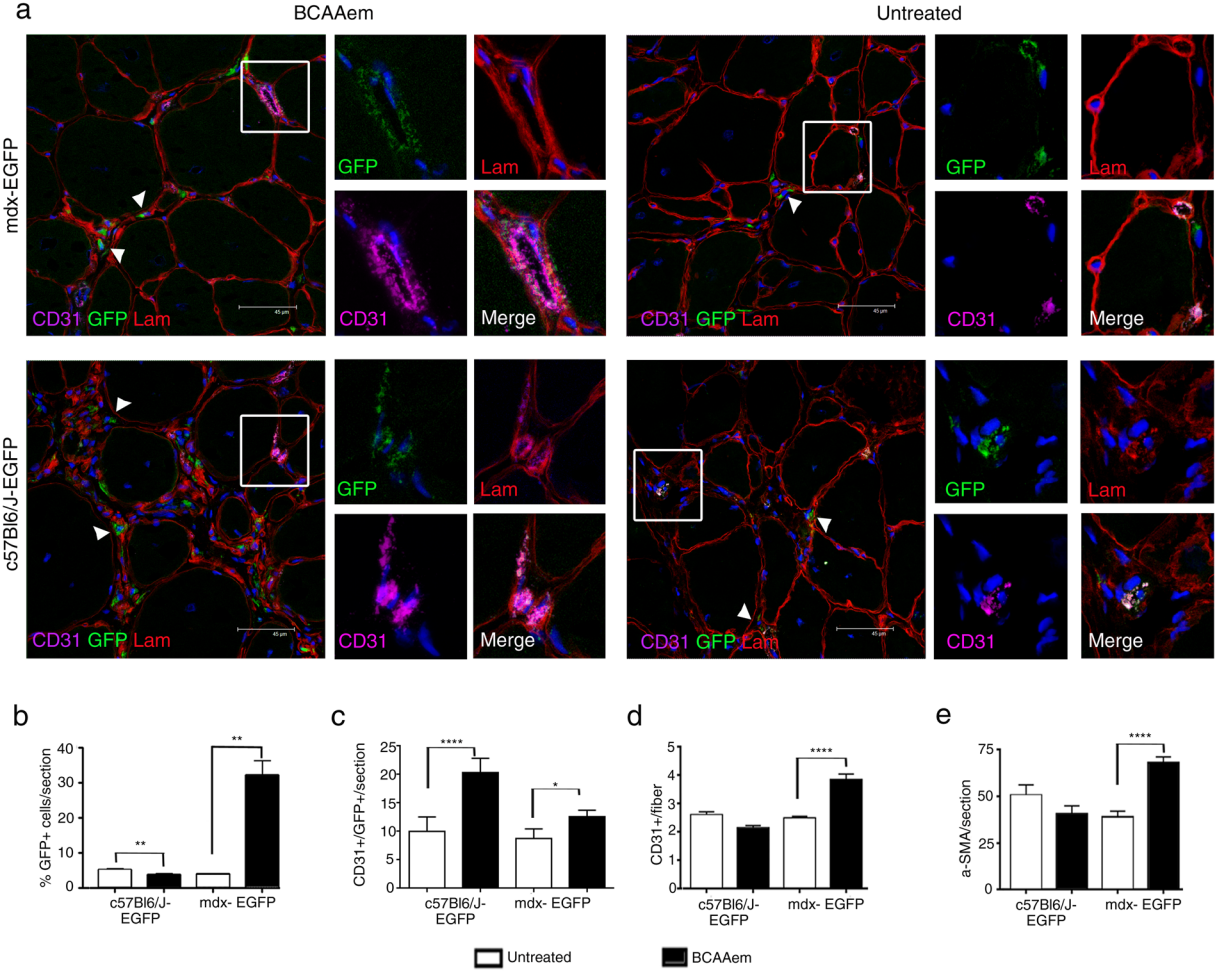
BCAAem recruits endothelial progenitors to healthy and dystrophic TA muscles. Cryosections of TA (**a**) muscle tissues of BCAAem-treated and untreated *C57BL6/J-*EGFP and *mdx-EGFP* mice were analysed by immunofluorescence staining for GFP, CD31 and laminin (Lam) (n = 5 for each experimental group). Nuclei were stained with DAPI. EGFP+ cells were detected in the interstitial spaces (arrowheads in a) and inside small CD31+ vessels (boxes in a). Magnification (X 1,000) of boxes indicate vessels expressing GFP (green), laminin (red), CD31 (purple). Merged images reveal vessel areas of costaining, as evidenced by the grey staining pattern. Analysis of the TA muscles of BCAAem-treated *mdx-EGFP* mice demonstrates an increase in the number of EGFP+ CD31+ and CD31+ vessels and α-SMA+ cells per section. Scale bar, 45 µm. The percentage of EGFP+ cells per section (**b**) and the number of EGFP+ CD31+ cells per section (**c**) are shown. The number of CD31+ vessels per fibre (**d**) and α-SMA+ cells per section (**e**) is shown. All data are presented as the mean ± s.e.m. Statistical error analysis was performed by one-way ANOVA with Bonferroni correction; *p < 0.05, **p < 0.01 and ****p < 0.0001 indicate comparisons that reflect significant differences relative to the untreated group.

**Figure 9:**
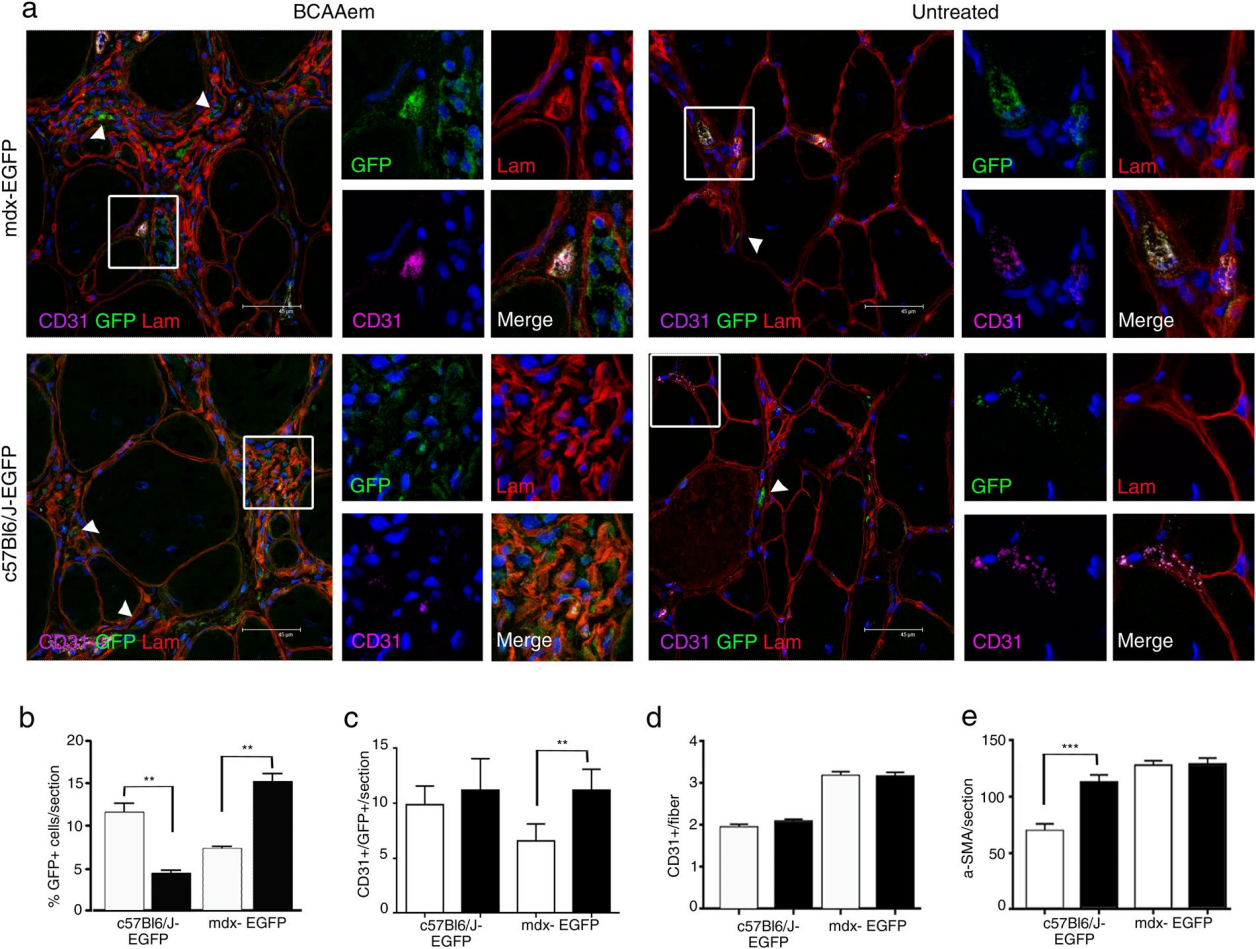
BCAAem recruits endothelial progenitors to dystrophic VM muscles. Cryosections of VM (**a**) muscle tissues of BCAAem-treated and untreated *C57BL6/J-EGFP* and *mdx-EGFP* mice were were immunostained for GFP, and costained for CD31 and laminin (lam) (n = 5 for each experimental group). Nuclei were stained with DAPI. EGFP+ cells were detected in the interstitial spaces (arrowheads in a) and inside small CD31+ vessels (boxes in a). Magnification (X 1,000) of boxes indicate vessels expressing GFP (green), laminin (red), CD31 (purple). Merged images reveal vessel areas of costaining, as evidenced by the grey staining pattern. Scale bar, 45 µm. Quantifications of EGFP+ cells (**b**) and EGFP+ CD31+ cells (**c**) in each section and CD31+ vessels per fibre (**d**) and α-SMA+ cells per section (**e**) are shown. All data are presented as the mean ± s.e.m. Statistical error analysis was performed by one-way ANOVA with Bonferroni correction; **p < 0.01 and ***p < 0.001 indicate comparisons that reflect significant differences relative to the untreated group. Histograms correspond to: untreated (white) and BCAAem-treated (black).

Next, we sought to identify the cellular mechanisms involved in the amelioration of skeletal muscle vascularization in dystrophic mice subjected to amino acid supplementation. Thus, the effect of BCAAem on the recruitment of circulating endothelial progenitors (EPs) to muscles was analysed^56^. We measured the number of SCA1+ CD34+ EPs in both muscle and blood of BCAAem-treated and untreated *C57BL6/J*, *mdx*, and *eNOS*^−/−^ mice. The number of SCA1+ CD34+ EPs was higher in the peripheral blood of BCAAem-treated mice of all genotypes (Fig. S4a; p < 0.0001). This result suggests that BCAAem supplementation promotes specific mobilization of SCA1+ CD34+ EPs from bone marrow (BM). Then, we analysed the presence of SCA1+ CD34+ EPs in the TA and VM muscles of untreated and treated animals (Fig. S4b, c). BCAAem treatment promoted a statistically significant increase in the number of SCA1+ CD34+ EPs in the muscles of wild-type *C57BL6/J* and dystrophic *mdx* mice (Fig. S4b,c, p < 0.05). *eNOS*^−/−^ mice did not exhibit any statistically significant increase in EP levels in the TA (Fig. S4b, p > 0.05) and even exhibited a statistically significant decrease in EP levels in the VM (Fig. S4c, p < 0.0001). To test the hypothesis that SCA1+ CD34+ EPs were present in the muscles of BCAAem-treated *C57BL6/J* and *mdx* mice due to mobilization of the EPs from BM, we performed BM transplantation experiments, followed by BCAAem treatment (1.5 mg/g body weight for 14 days). Lethally irradiated *C57BL6/J* (n = 10) and *mdx* (n = 10) mice were transplanted with BM cells isolated from wild-type *EGFP* mice. BM engraftment efficiency was tested six weeks after the transplant, evaluating by FACS the percentage of EGFP+ cells in peripheral blood. The percentage of circulating EGFP+ cells in transplanted animals was >75% of the total cells in both the *C57BL6/J* and *mdx* genotypes. Hereafter, the EGFP BM-transplanted animals are referred as *C57BL6/J-EGFP* and *mdx-EGFP*. After 8 weeks, the time needed for long-term BM reconstitution, these animals were supplemented or not with BCAAem (n = 5 per group) and sacrificed after an additional 15 days. We found a statistically significant increase in the number of SCA1+ CD34+ EGFP+ EPs in the blood and skeletal muscles of BCAAem-treated *C57BL6/J-EGFP* and *mdx-EGFP* mice compared to the number of such EPs observed in the untreated controls (Fig. 7a–c, p < 0.05). To elaborate, we observed a ~10-fold increase in the number of SCA1+ CD34+ EGFP+ EPs in the TA and VM muscles of BCAAem-treated *mdx-EGFP* mice compared with the number of such EPs in untreated mice (untreated 0.033% *vs*. treated 0.375% in TA; untreated 0.03% *vs*. treated 0.2% in VM; p < 0.05). Moreover, the number of CD31+ EGFP+ and CD90+ EGFP+ ECs increased in the blood and muscles of BCAAem-treated *mdx-EGFP* mice (Fig. 7a–c). Together, these data strongly suggest that BCAAem supplementation stimulates the muscle homing of EPs and the muscular levels of ECs in dystrophic mice. To further support the hypothesis that BCAAem promotes EP migration from BM to skeletal muscles, the key chemotactic system that promotes angiogenesis was investigated. CXC chemokine receptor 4 (CXCR4) is an alpha-chemokine receptor that is specific for stromal cell-derived factor 1 (SDF-1), which is exhibits potent chemotactic activity in various cell types. Moreover, the SDF-1/CXCR4 interaction was found to contribute to the regulation of EP recruitment in ischaemic and damaged muscle tissues^57,58^. We observed a statistically significant increase in the levels of CXCR4+ EGFP+ cells, mobilized from the transplanted BM, and SDF1+ EGFP- resident cells in the TA muscles of BCAAem-treated *mdx-EGFP* and BCAAem-treated *C57BL6/J-EGFP* mice (Fig. 7d, p < 0.0001). Based on the muscular vessel amelioration observed in BCAAem-treated *mdx* mice, we identified a statistically significant increase in the number of CD31+ vessels per muscle fibre and in the level of α-SMA+ cells (two-fold increase, p < 0.0001) in the TA muscles of BCAAem-treated *mdx-EGFP* mice (Fig. 8). Notably, EGFP+ cells were found in the interstitial space and inside small CD31+ vessels (Figs 8 and 9). We found that EGFP+ cells had incorporated into the endothelium of small caliber vessels as revealed by coexpression of GFP and endothelial marker CD31 (Figs 8 and 9). The merger of GFP and CD31 staining patterns reveals colocalization (grey color) in vessel structures (Figs 8 and 9). The number of EGFP+ cells that exhibited CD31 was significantly higher in TA and VM muscles of BCAAem-treated *mdx-EGFP* (12,80 ± 0,58/section and 11,20 ± 0,86/section *vs*. untreated 8,75 ± 0,85/section and 6.6 ± 0,67/section respectively; p < 0.05 and p < 0.01 one-way ANOVA Bonferroni) and in TA muscle of BCAAem-treated *C57BL6/J-EGFP* mice (20,30 ± 1,45/section *vs*. untreated 10,0 ± 1,14/section; p < 0.0001 one-way ANOVA Bonferroni). No significant differences were observed between VM muscle of BCAAem-treated and untreated *C57BL6/J-EGFP* mice (11,27 ± 1,28/section *vs*. untreated 9,9 ± 0,74/section). The localization of EGFP+ cells confirms the effective presence in circulating blood of BCAAem-mobilized cells and colonization of the vessel walls by these cells. Histological and biochemical analyses of the TA and VM muscles of BCAAem-treated *mdx-EGFP* mice showed amelioration of the dystrophic muscle phenotype in terms of mean CSA values in the VM muscles (BCAAem-treated *vs*. untreated, 2203 ± 37 μm^2^ vs. 2704 ± 43 μm^2^, p < 0.0001) (Fig. S5). A similar trend was observed in the TAs of *mdx-EGFP* mice (BCAAem-treated *vs*. untreated, 2010 ± 32 μm^2^ *vs*. 2061 ± 32 μm^2^) (Fig. S5). Furthermore, the endothelial differentiation potential of BM-derived EPs isolated by FACS from BCAAem-treated *mdx-EGFP* mice was statistically higher than that of EPs obtained from untreated *mdx-EGFP* mice (p < 0.0001 one-way ANOVA Bonferroni) (Fig. S6). Taken together, these findings suggest that BCAAem supplementation induces the recruitment of BM-derived EPs in the skeletal muscles of *mdx* mice via the SDF-1-CXCR4 axis.

### Amelioration of muscle dysfunction in *mdx-EGFP* mice subjected to BCAAem supplementation

BCAAem-treated and untreated *mdx-EGFP and C57BL6/J-EGFP* mice were tested for motor capacity, specific force, and fatigue index as previously described. BCAAem-treated *mdx-EGFP* mice showed statistically significant enhancement of time to exhaustion and endurance compared to untreated *mdx-EGFP* mice (Fig. S7). In these experiments, the running performance of the BCAAem-treated *mdx-EGFP* mice was approximately 75% higher than that of the untreated mice (Fig. S7). Statistically significant differences were observed in the fatigue indices of BCAAem-treated and untreated *mdx-EGFP* mice (Fig. S7).

## Discussion

In this study, we have reported that dietary supplementation with a specific amino acid formulation (BCAAem) induces an increase in oxidative muscle fibre content and has an angiogenic effect in the muscles of *mdx* mice to ameliorate dystrophic symptoms, especially in females. *Mdx* is a murine model of DMD that is characterized by pathological similarities to the human disease^59–62^. BCAAem enhances the oxidative myofibre content of skeletal muscles and increases eNOS activation (p-eNOS) and ERα and VEGF expression. These processes are accompanied by muscle recruitment of EPs, which ameliorates capillary density in skeletal muscles and increases the endurance time of BCAAem-treated dystrophic *mdx* mice. The major clinical symptoms of DMD are caused by dystrophin loss, which leads to sarcolemmal fragility and consequent muscle wasting^63^. DMD remains an incurable muscular disorder, and the proposed replacement of the dystrophin gene is associated with multiple technical problems and only few and limited positive results. Furthermore, the signalling pathways involved in DMD, especially the regulation of oxidative metabolism in dystrophic skeletal muscle, are far from being well understood. Glycolytic muscle fibres are more susceptible to dystrophic damage, thus therapeutic strategies to increase oxidative fibre content have been proposed for DMD^17–20^. Our present findings demonstrate that dietary BCAAem consumption for 15 consecutive days reduces the extreme CSA distribution of the muscle fibres of *mdx* mice, suggesting that a change in muscular metabolism occurs. We analysed the fibre composition of the skeletal muscle via three different approaches, demonstrating that BCAAem induces an increase in oxidative myofibre content, with MyHC IIx expression in both the VM and TA muscles of BCAAem-treated *mdx* male and female mice. These findings are consistent with those reported in previous studies by us and other researchers^42,64^ on the ability of BCAAem to upregulate PGC-1α and SIRT1 expression and activity in skeletal muscle and on the roles of PGC-1α and ERα as key transcriptional regulators of the change in fibre type in skeletal muscle. Skeletal muscle, in fact, is an oestrogen-responsive tissue that expresses high levels of ERα mRNA. ERα drives muscle regenerative capacity, the expression of MyHC isoforms and resistance to fatigue^65^. Furthermore, the described PGC-1α-dependent changes in myofibre composition are mediated by the oestrogen receptor^66,67^. Accordingly, we first demonstrated a stronger and more consistent change in MyHC IIx expression in female BCAAem-treated *mdx* mice than in male BCAAem-treated *mdx* mice, with significantly higher expression of ERα. Since ERα stimulation also promotes vessel remodelling via eNOS expression^42,54^, we tested the hypothesis that the BCAAem-induced change in fibre type is accompanied by an angiogenic muscle response. The absence of dystrophin in DMD causes nNOS displacement from the sarcolemmal membrane, and the consequent reduction of NO production causes vasoconstriction and abnormal blood flow during skeletal muscle contraction^25^. Moreover, dystrophin normally forms a complex with eNOS in ECs, and consequently, in *mdx* mice, NO production is markedly compromised in ECs, with impaired angiogenesis observed in muscles^68^. Accordingly, a reduced number of CD31+ vessels were observed in the muscles of dystrophic *mdx* mice compared with the number observed in healthy *C57BL6/J* mice, and notably, BCAAem supplementation increased the number of CD31+ vessels in the muscles of *mdx* mice, unlike what was observed in *eNOS*^−/−^ mice. This effect was accompanied by consistent induction of eNOS and VEGF expression in the muscles of BCAAem-treated *mdx* mice. Furthermore, we have shown *in vivo* that BCAAem ameliorates the compromised vessel architecture in BCAAem-supplemented *mdx* mice by increasing the number of CD31/α-SMA and CD31/VE-cad double-positive vessels. These results suggest progressive maturation of the blood vessels and normalization of the capillary walls. Recently, higher expression of the eNOS protein was observed in the ECs of females than in those of males^69^, suggesting intrinsic sexual dimorphism of these cells. Accordingly, we demonstrated a greater increase in the number of CD31+ vessels in female BCAAem-treated *mdx* mice than in male BCAAem-treated *mdx* mice. These data, together with the stronger induction of VEGF and eNOS activation in female mice than in male mice, suggest that the differential response to BCAAem supplementation in terms of angiogenesis induction could be linked to a higher capacity of female muscle tissue to respond to BCAAem treatment due to sex-related differences. Consistent with the positive effects on muscle performance demonstrated by the increase in oxidative fibre content in mice^70–72^, we observed an increased fatigue resistance in BCAAem-treated *mdx* mice. In addition, the increased vascular density and maturation observed in BCAAem-treated *mdx* mice are consistent with the improvement of the muscle performance of dystrophic mice, as previously reported^73^. To understand how BCAAem induces muscular vessel remodelling, we analysed the EP levels in the blood and muscles of healthy *C57BL6/J*, dystrophic *mdx*, and *eNOS*^−/−^ mice. Interestingly, we found that the number of CD34+ SCA1+ EPs was higher in BCAAem-supplemented mice of all genotypes. In contrast, the number of CD34+ SCA1+ EPs was higher in the muscles of *mdx* mice but not *C57BL6/J* and *eNOS*^−/−^ mice, which leads us to hypothesize that dystrophic muscle damage may influence the degree of vessel remodelling in response to BCAAem. Moreover, the absence of structural and density modifications of vessels in *eNOS*^−/−^ mice further confirmed the key role of eNOS in mediating vessel remodelling in response to BCAAem treatment. To identify the origins of the EPs in dystrophic muscle tissues, *EGFP-C57BL6/J* BM was transplanted into *C57BL6/J* and *mdx* mice before amino acid supplementation. BCAAem promoted a statistically significant increase in the number of EGFP+ SCA1+/CD34+ EPs in the TA and VM skeletal muscles of dystrophic mice compared to the number observed in *C57BL6/J* healthy animals. Moreover, the number of EGFP+ CD31+ and CD90+ ECs in the skeletal muscles was significantly higher in BCAAem-treated mice than in untreated *mdx* mice. Interestingly, the number of CD31^+^ capillaries per fibre in the muscles of BCAAem-treated and untreated BM-transplanted mice was higher than the number observed for non-transplanted mice, suggesting that donor BM endothelial progenitor cells were directly incorporated into the host vessels, thereby forming a mosaic pattern of BM progenitor-derived and locally derived CD31^+^ capillaries. Additionally, BCAAem may greatly influence the promotion of the muscle homing of BM EPs. These results were reinforced by the high number of circulating EPs (EGFP+ CD34+/SCA1+) and ECs (EGFP+ CD90+ and EGFP+ CD31+) in BCAAem-treated mice of all genotypes, suggesting the occurrence of EP mobilization from BM with subsequent accumulation in skeletal muscles. Muscle homing of EPs was confirmed by the increased number of circulating EGFP+ CXCR4+ cells and concomitant expression of SDF-1 in the skeletal muscles of BCAAem-treated *mdx* mice. The angiogenic potential of circulating EGFP+ EPs of BCAAem-treated *mdx* mice was also experimentally confirmed by showing the capacity of these EPs to form endothelial colonies *in vitro*. These results suggest that the observed mobilization of EPs from BM to dystrophic skeletal muscle may depend on the muscular levels of VEGF, which may act as a regulator of SDF-1^66,67,74^, and these results are consistent with previous reports^25,27,73,75^ showing the role of the SDF1/CXCR4 axis in the muscle recruitment of EPs. Overall, our data suggest that dietary supplementation with BCAAem could be an effective adjuvant treatment for human DMD because BCAAem changes the energy metabolism of muscles (i.e., increases oxidative muscle fibre content), ameliorates vascularization (i.e., increases capillary density) of skeletal muscle, and increases muscle fatigue resistance in *mdx* mice.

## Methods

### Ethics statement and experimental model details

Procedures involving live animals conformed to Italian law (D.L.vo 116/92) and were subsequently approved by ethics committees. The *in vivo* experiments were authorized by the Ethical Committee of Università degli Studi di Milano, protocol number 10/13–2014/2015. Three-month-old normal (*C57BL6/J*), dystrophic (*mdx C57BL6/*10ScSn-DMD*mdx*/J), *eNOS*^−/−^ (B6.129P2-Nos3^tm1Uncmice^/J) and EGFP/C57BL6/J mice were all provided by Jackson Laboratory (USA). The weight of each *C57BL6/J* mouse was approximately 19 gr, while the weight of each *mdx* mouse was 25 gr. Animals were treated humanely, and animal cages were maintained in controlled ambient light (12-hour light, 12-hour dark) at temperatures between 21 °C and 24 °C. The animals were able to move freely within the cages and had access to clean water and food. After 15 days of treatment with the BCAAem mixture, mice were sacrificed by cervical dislocation according to Italian law. For surgical procedures, mice were anaesthetized with 2% Avertin (0.015 ml/kg body weight) by intra-peritoneal injection.

### BCAAem mixture composition, dosage and absorption

BCAAem supplementation was performed for two weeks. BCAAem (1.5 mg/g body weight/day in drinking water, with percent composition of the mixture as previously detailed)^42,76^ was dissolved in tap water and stored at 4 °C before daily administration. Drinking water was provided in a graduated bottle placed on top of each cage, in which 2 mice were housed. The drinking bottle was connected to a sipping tube containing a metal ball to prevent leakage of water. The volume of water was measured daily throughout the treatment. The average volume of water consumed per mouse per day was calculated. Data were expressed as ml/animal/day. We measured the circulating BCAA (leucine, isoleucine, and valine) levels in wild-type (*C57BL6/J*) and *mdx m*ice acutely (30–120 min) supplemented with BCAAem as previously described by D’Antona *et al*.^42^.

### Immunohistochemistry and immunofluorescence

VM and TA muscles were removed from both the treated and untreated mice of each genotype described, frozen in liquid-nitrogen-cooled isopentane, embedded in Tissue-Tek® O.C.T. compound (Sakura Finetek, USA) and sectioned in 10-mm-thin sections with a CM 1820 cryostat (Leica, Germany). Histological analyses were performed on the VM and TA muscles as these muscles show similar levels of dystrophic degeneration in *mdx* mice. Serial sections were stained with Haematoxylin and Eosin (H&E) and Azan-Mallory (AM) stain. Images were captured with a Leica DM6000 B (Leica, Germany) optical microscope. Quantification of fibre area was performed manually using NIH ImageJ software 6.0. Myofibrillar ATPase staining with pre-incubation at pH 4.3 was used to identify fibre types IIa, IIx and IIb in TA and VM muscles. ATPase (pH 4.3) staining was performed as described in recent publications^77–79^. After staining, the sections were washed with several changes of tap water, dehydrated with ethanol, cleared in xylene, and mounted in glycerol-gelatin aqueous slide mounting medium (Sigma-Aldrich, USA). SDH staining was performed in accordance to Bloemberg *et al*.^80^. Images were captured with a Leica AS LMD optical microscope. Quantification of different fibre types was performed using ImageJ software (NIH); 32-bit images were analysed by histogram distribution of grey values in the muscle-selected area, presented as pixel counts for each grey value. Values were then grouped in specific intervals according to different fibre types. For immunofluorescence analysis, sections were fixed in 80% ethanol or 100% cold acetone and incubated with the following antibodies: rabbit anti-laminin (1:100; Sigma, Germany), rat anti-CD31 (1:50; BD, USA), anti-smooth muscle actin (1:50; Sigma-Aldrich, USA), rabbit anti-von Willebrand factor (vWF) (1:400; Dako, Denmark), mouse anti-VE-cadherin (1:50; Chemicon International, USA), mouse anti-MyHC fast (1:20; Hybridoma Bank, Stanford, California, USA) and mouse anti-MyHC slow (1:20; Hybridoma Bank, Stanford, California, USA). The sections were rinsed in phosphate-buffered saline (PBS) and incubated with the corresponding Alexa Fluor 488, Alexa Fluor 594 or Alexa Fluor 647-conjugated secondary antibodies (1:100; Molecular Probes, Thermo Scientific, USA). To detect EGFP+ cells, slides were incubated with rabbit anti-GFP antibody (1:100; Molecular Probes, Thermo Scientific, USA). Cell nuclei were stained with 4′,6-diamidino-2-phenylindole (DAPI). Images were captured with a Leica TCS-SP2 confocal microscope. For quantitative analysis, NIH ImageJ software 6.0 was used. Vessel count was performed by evaluating the number of α-SMA+ vessels in at least three sections for each slide, while CD31+ capillaries were evaluated by acquiring at least seven images for each slide and by calculating the ratio between CD31+ capillaries and fibres.

### Probe-based confocal laser endomicroscopy (p-CLE)

CD31 Alexa Fluor 488, VE-cad Alexa Fluor 647 (BioLegend, San Diego), and α-SMA Alexa Fluor 647 (Abcam, UK) (1 μg/g body weight) were injected via the tail vein, and the mice were shielded from light. Endomicroscopic imaging was performed 24 hours later with a Cellvizio Dual Band endomicroscopy system (Mauna Kea Technologies) according to the manufacturer’s instructions, and images were acquired with Cellvizio software^81^. Mice were anaesthetized with 2% Avertin (0.015 ml/kg body weight), and muscles were exposed for CLE by a 3-mm skin incision. The confocal probe was inserted into the TA muscle to scan the tissue. Several incisions were then made to investigate deeper parts of the tissue. Mice were euthanized prior to recovery from anaesthesia.

### *In vivo* muscle function assessment

Exercise capacity was assessed using an incremental treadmill exhaustion test, as previously described^42^. Briefly, each animal was placed on the belt of a 6-lane motorized treadmill (Exer 3/6 treadmill, Columbus Instruments, OH, USA) supplied with shocker plates, which could be individually enabled or disabled for each lane. During each exhaustion test, the duration of the electrical stimulus was fixed at 200 ms, with a 0.34-mA amplitude, and 1-Hz repetition rate. After acclimatization, all mice were subjected to the initial treadmill exhaustion tests at a 0° inclination according to the following protocol: 5 min at 5 m/min, followed by an incremental increase in speed of 1 m/min until exhaustion. Exhaustion was achieved when the mice stayed on the shocker plate without attempting to reengage the treadmill for 20 s or more. Three tests were performed on each animal, allowing 4 days between each test. The values were averaged to obtain a single value per animal. In a second set of experiments, endurance was evaluated as the time spent on the treadmill belt running at 50% of the maximal velocity reached in the previous incremental exhaustion test (endurance time).

### *Exvivo* muscle function assessment

The method used for mechanical analysis of intact muscles has been previously described^47^. Briefly, the TA muscle of the right leg was dissected and placed in an organ bath filled with Krebs solution (120 mM NaCl, 2.4 mM KCl, 2.5 mM CaCl_2_, 1.2 mM MgSO_4_, 5.6 mM glucose, 1.2 mM KH_2_PO_4_, and 24.8 mM NaHCO_3_; pH 7.4). The bath was bubbled with 95% O_2_ and 5% CO_2_ at a constant temperature of 22 °C and attached to a force transducer (Radnoti organ bath system, AD Instruments). Electrical pulses were delivered through platinum electrodes connected to a stimulator (Tumiati, Italy). Tetanic isometric contractions were evoked (110 Hz, 500 ms, supramaximal amplitude) at the length at which the maximal isometric force was observed, and the twitch time to peak and maximal tetanic force (Tf) values were measured. Specific tetanic force (i.e., maximal tetanic force normalized to muscle volume; g/μL) was considered for comparisons among the different groups of animals. Further, the fatigue index was measured (and expressed in %) as the decrease in tetanic force at different stimulation frequencies (0.03, 0.09, and 0.3 Hz) compared to the maximal tetanic force^47^.

### RT-PCR analysis

Total RNA was extracted from the muscles of male and female *mdx* mice with TRIzol (Thermo Scientific, USA) according to the manufacturer’s instructions. First-strand cDNA was prepared as previously described^82^. The following primers were used: MYH1-Fw 5′-ggaggaggaaatcgaggcag-3′, Rev 5′-cgctgatctcctccagttcc-3′; MYH2b-Fw 5′-gggtctgaactctgctgacc-3′, Rev 5′-tggcctttggtgacgtactc-3′; MYH4-Fw 5′-tgacgaccttgagctgacac-3′, Rev 5′-gggccttcttctccttggtc-3′; GADPH-Fw 5′-caaggctgtgggcaaggt-3′, Rev 5′-ggcaggtcagatccacaactg-3′. The expression levels of each gene were measured using SYBR Green (GoTaq PCR Master Mix; Promega, Madison, USA). Analyses were performed on duplicate cDNA samples from different muscle samples. Threshold cycle (Ct) values of target genes were normalized against that of the housekeeping gene glyceraldehyde 3-phosphate dehydrogenase (*GAPDH*), and relative transcript levels were calculated from the Ct values as X = 2^−Δct^, where X is the fold difference in amount of target gene versus the amount of *GAPDH*, and ΔCt = Ct_target_ − Ct_GAPDH_.

### Bone marrow transplantation

Recipient mice were lethally irradiated at 8.5 Gy 20 hours before transplant. Under sterile conditions, bone marrow cells (BMCs) were isolated by smashing the femurs of *EGFP/C57BL6/J* mice in PBS and were purified by Histopaque®-1077 (Sigma-Aldrich, USA) according to manufacturer’s instructions. BMCs were pooled from at least 3 independent donors, and 1.5 × 10^6^ cells were injected into the tail veins of lethally irradiated mice, either *C57BL6*/J or *C57BL6/*10ScSn-DMD*mdx*/J. The irradiation efficiency was monitored without reconstituting one mouse for each strain. These sentinels died after 15 days. At sacrifice, the percentage of EGFP+ cells in blood and TA/VM muscles was evaluated by FACS, and the cells were analysed for co-expression of Sca-1, CD34, CD31, CD90, CXCR4 and SDF-1.

### Western blot analysis

BMCs were lysed in RIPA buffer (50 mM Tris-HCl (pH 7.4), 1% NP-40, 0.25% Na-deoxycholate, 150 mM NaCl, 1 mM EDTA). Muscle samples were lysed in RIPA buffer with a tissue homogenizer. Total protein concentrations were determined by the BCA assay, and measurements were made with a Glomax microplate luminometer (Promega, USA). Samples were resolved on an 8% polyacrylamide gel and transferred to supported nitrocellulose membranes (Bio-Rad Laboratories). Next, the membranes were blocked in 5% milk dissolved in TBS-T (20 mM Tris, 150 mM NaCl and 0.1% Tween 20). Then, the membranes were incubated overnight at 4 °C with primary antibodies against the following targets: p-eNOS (Ser1177) (cod 9571, Cell Signaling, USA), eNOS (cod 9572, Cell Signaling, USA), ERRα (cod sc-787, Santa Cruz, USA), VEGF (cod sc-507, Santa Cruz, USA), and vinculin (Sigma-Aldrich, USA). Then, the membrane was washed extensively with TBS-T, probed with horseradish peroxidase (HRP)-conjugated secondary antibodies (Dako, USA) and developed with ECL (Amersham Biosciences, Italy). Each experiment was performed on at least three different mice for each experimental condition. Densitometric analyses were performed using NIH ImageJ software.

### Evaluation of myosin heavy chain (MyHC) isoforms

Muscle samples were lysed in SDP buffer (10% sodium dodecyl phosphate, 40 mM dithiothreitol (DTT), 5 mM EDTA, 0.1 M Tris (pH 8)) with a tissue homogenizer. Total protein concentration was determined by the BCA assay and quantified with a Glomax microplate luminometer (Promega, USA). Samples were resolved on a 6% polyacrylamide-30% glycerol gel at 4 °C for 24 hours and subjected to standard Coomassie Blue staining, and images were acquired with an Odyssey® imaging system (LI-COR, Nebraska, USA) and Image Studio Lite version 5.2. Densitometric analyses were performed with NIH ImageJ software. Each experiment was performed on at least three different mice for each experimental condition.

### Statistics

For histograms, data are expressed as the mean ± s.e.m. In the box and whisker plots, boxes indicate 25^th^ to 75^th^ percentiles; whiskers indicate 5^th^ to 95^th^ percentiles; and the line indicates the median. To compare the means of multiple groups, one-way or two-way ANOVA was used in the Prism 6 software (GraphPad software, La Jolla, CA) with Bonferroni correction. The difference among groups was considered significant at p < 0.05.

## Electronic supplementary material

Supplementary information
