# Supplementary material for: Supplementation with a selective amino acid formula ameliorates muscular dystrophy in mdx mice
Source: Sci Rep. 2018 Oct 2;8:14659. doi: 10.1038/s41598-018-32613-w (PMC6168581; doi:10.1038/s41598-018-32613-w)
Supplement: Supplementary file 1 — Supplementary information [file 41598_2018_32613_MOESM1_ESM.pdf]

SUPPLEMENTARY MATERIAL

Supplementation with a selective amino acid formula ameliorates muscular dystrophy in *mdx* mice

Stefania Banfi,[1] Giuseppe D'Antona,[2] Chiara Ruocco,[3] Mirella Meregalli,[1] Marzia Belicchi,[1] Pamela Bella,[1] Silvia Erratico,[4] Elisa Donato,[5,6] Fabio Rossi,[3] Francesco Bifari,[7] Caterina Lonati,[8] Stefano Campaner,[5] Enzo Nisoli,[3,*] Yvan Torrente,[1,*]

[1]Department of Pathophysiology and Transplantation, Università degli Studi di Milano, Fondazione IRCCS Ca' Granda Ospedale Maggiore Policlinico, Centro Dino Ferrari, 20122 Milan, Italy

[2]Department of Public Health, Molecular and Forensic Medicine, and Sport Medicine Centre Voghera, University of Pavia, Pavia, 27100, Italy

[3]Center for Study and Research on Obesity, Department of Medical Biotechnology and Translational Medicine, University of Milan, Milan, 20129, Italy

[4]Novystem, Milan, 20129, Italy

[5]Centre for Genomic Science of IIT@SEMM, Istituto Italiano di Tecnologia, Milan, 20139, Italy

[6]Present address: Division of Stem Cells and Cancer, Deutsches Krebsforschungszentrum, Heidelberg, Germany and Heidelberg Institute for Stem Cell Technology and Experimental Medicine, Heidelberg, Germany

[7]Laboratory of Cell Metabolism and Regenerative Medicine, Department of Medical Biotechnology and Translational Medicine, University of Milan, Milan, 20129 Milan, Italy

[8]Center for Surgical Research, Fondazione IRCCS Ca' Granda, Ospedale Maggiore Policlinico, Milan, 20122, Italy

*Correspondence: yvan.torrente@unimi.it (Y.T.), enzo.nisoli@unimi.it (E.N.)

SUPPLEMENTARY METHODS

FACS analysis.

Peripheral blood (100 µl) from the retro orbital sinus was collected from each mouse, and red blood cells were lysed with ACK solution (150 mM NH_4Cl, 10 mM $KHCO_3$ and 0.1 mM Na_2EDTA) to allow cytofluorimetric studies. For five-colour flow cytometry, cells were incubated with 10 µl of primary antibodies against CD34 FITC, SCA1 PE (BD Biosciences, San Diego, CA, USA), CD184 (CXCR4) APC (Miltenyi Biotech, Bologna, Italy), CD31 (PECAM-1) PE (BD Biosciences, San Diego, CA, USA), CD90 (Thy-1) PE (BD Biosciences, San Diego, CA, USA), and SDF-1 (CXCL12 R&D systems), which was followed by incubation with secondary goat anti-mouse antibody (Alexa Fluor 647, Thermo Fisher Scientific). Muscles were weighed and washed several times in PBS, finely minced with scissors, and incubated at 37°C for 45 min with 1 mg/mL collagenase type I (Sigma-Aldrich, USA), 80 g/mL DNase I (Roche), and 2.5% trypsin (1:3) (Gibco) in Dulbecco's modified Eagle's medium (DMEM) (Invitrogen). Most of the skeletal muscle stem cells were released from the tissue after this step. The cell extract was filtered with a 70-µm nylon mesh (BD Biosciences, Immunocytometry Systems, Mountain View, CA) and labelled for FACS analysis as described above. After each incubation, performed at 4°C for 20 min, cells were washed in PBS containing 1% heat-inactivated foetal calf serum (FCS) and 0.1% sodium azide. Isotype-matched immunoglobulins were added to each control sample. The cells were analysed using a Cytomics FC500 instrument and CXP 2.1 software (BC, Beckman-Coulter). Each analysis included at least 50000–200000 events for each gate. A light-scatter gate was set to eliminate cell debris from the analysis. The percentage of positive cells was assessed after correction for the percentage of cells reactive to an isotype control conjugated to relative fluorochromes. Bone marrow was collected from treated and untreated *mdx* and *C57BL6/J* mice by flushing

femurs and tibias with saline solution, and red blood cells were lysed with ACK solution. Nucleated cells were labelled in PBS containing 2% FCS for 45 min at 4°C with the previously described antibodies.

Endothelial colony formation assay.

Blood-circulating EGFP+ and EGFP- cells were isolated by FACS for co-expression of Sca1 and CD34 and incubated in an endothelial growth medium containing medium 199 (Gibco-BRL, Life Technologies, Thermo Fisher Scientific) supplemented with 50 ng/ml VEGF. Cells were then seeded at a density of 30,000 cell/cm^2 on six-well plates coated with Matrigel (Corning, Corning, NY, https://www.corning.com/medium)/199 medium (1:1) and incubated in a humidified atmosphere with 5% CO_2/95% air, at 37°C. For each condition, cells from at least three different mice were seeded in triplicate wells. After 24–48 hours, we observed the formation of colonies by using an inverted microscope (Leica DMIRE2). To evaluate endothelial colony formation potential, three different operators counted colonies with more than 20 cells (Mandò et al., 2016).

SUPPLEMENTARY FIGURES AND FIGURE LEGENDS

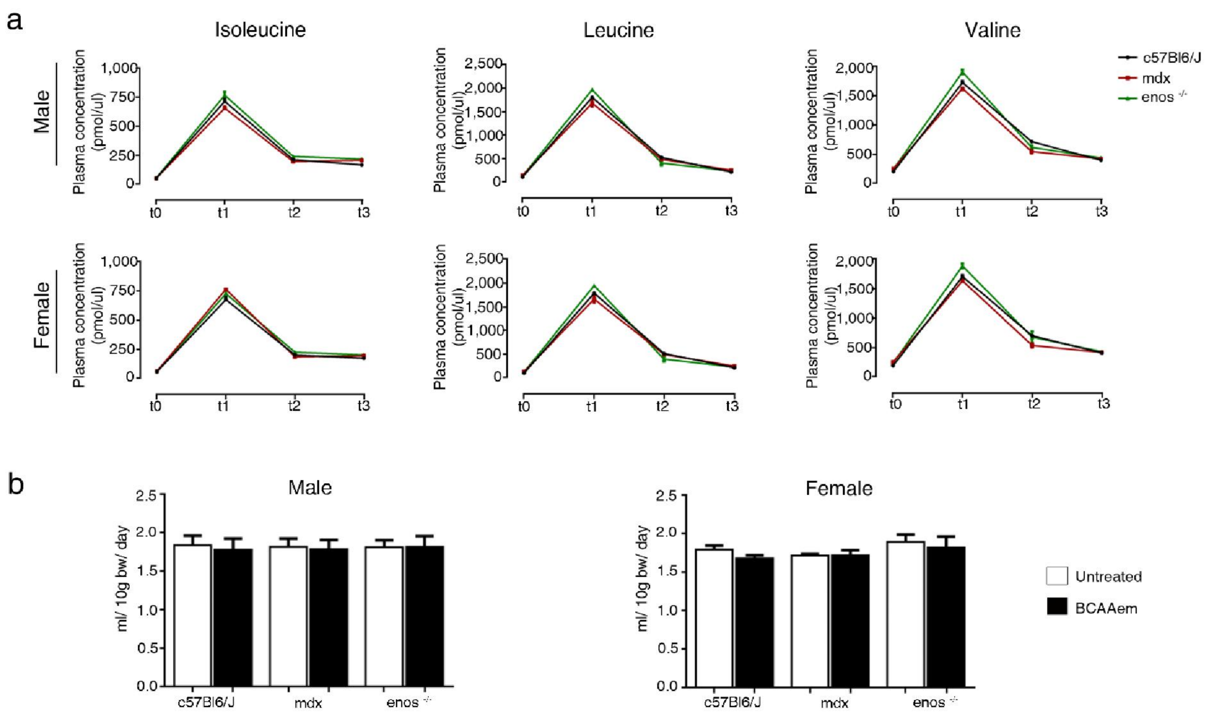

Supplementary Figure 1

Changes in plasma branched-chain amino acids after BCAAem bolus administration and evaluation of daily drinking water

Plasma samples of untreated male and female *C57BL*6/J (n=4) and *mdx* (n=4) mice were obtained before (t0) and at different time intervals after (t1, 30 min; t2, 60 min; t3, 120 min) a single bolus of BCAAem, corresponding to the daily supplementation dose (1.5 g/kg body weight), was dissolved in tap water and administered by gavage (**a**). Water consumption was measured daily (**b**). Data are expressed as the mean ± s.e.m. Statistical error analysis was performed by two-way ANOVA with Bonferroni correction.

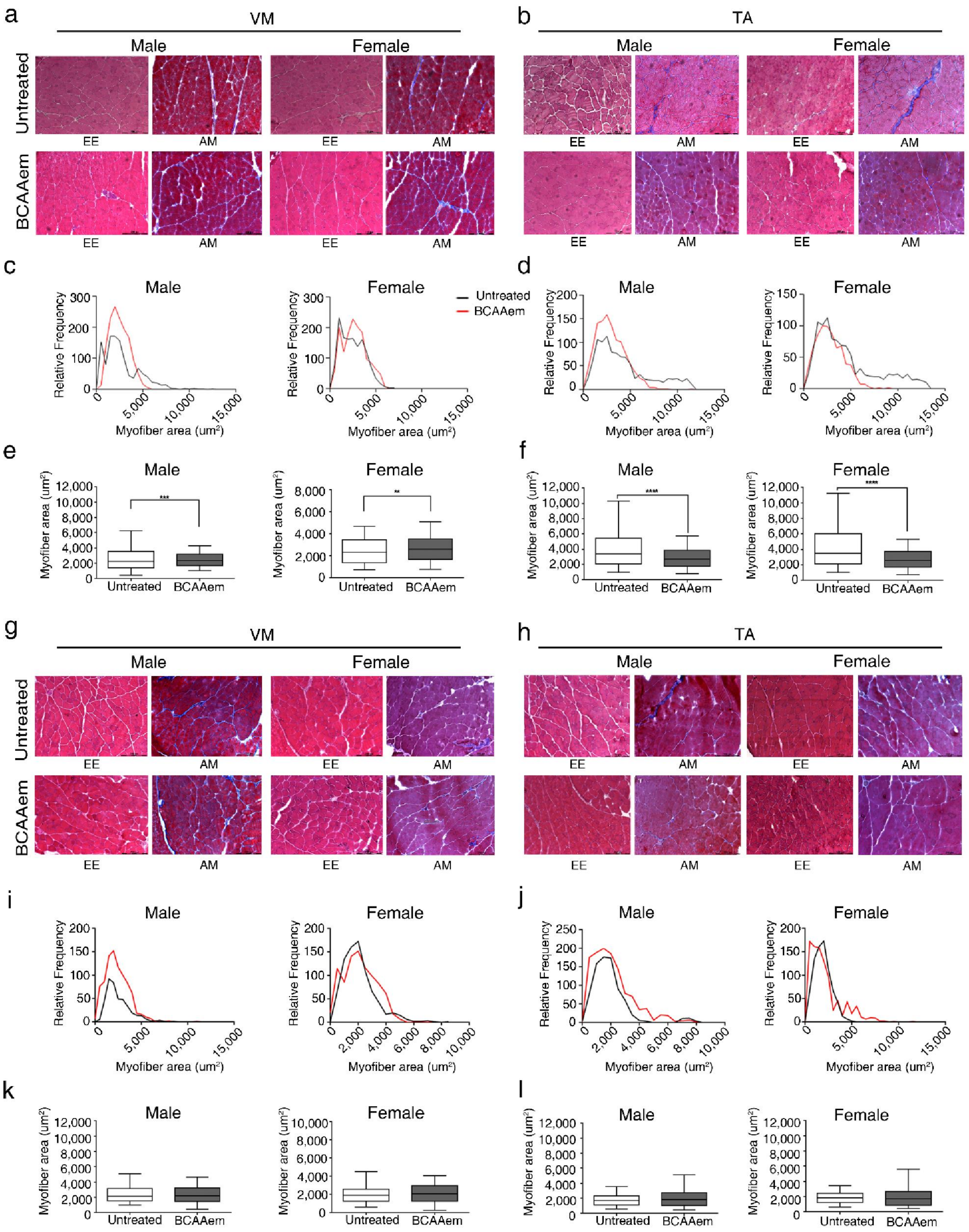

a
VM
Male
Female
Untreated
BCAAem
EE
AM
b
TA
c
Male
Female
Relative Frequency
Myofiber area (um²)
Untreated
BCAAem
d
e
Myofiber area (um²)
Untreated
BCAAem
***
**
f
****
g
h
i
j
k
l

Supplementary Figure 2

Haematoxylin and Eosin (H&E) and Azan-Mallory (AM) analysis of BCAAem-treated and untreated male and female *C57BL6/J* and *eNOS*$^{-/-}$ mice. Representative H&E and AM staining of the *vastus medialis* (VM) (**a and g**) and *tibialis anterior* (TA) (**b and h**) of untreated and BCAAem-treated male and female *C57BL6/J* **(a and b)** and *eNOS*$^{-/-}$ **(g and h)** mice. Scale bars, 200µm. Quantification of the relative frequency of the myofibre cross-sectional area (CSA) expressed as the frequency distribution of the VM (**c, e, i and k**) and TA (**d, f, j and l**) muscles from each of the untreated and treated male and female subgroups of *C57BL6/J* **(a-f)** and *eNOS*$^{-/-}$ **(g-l)** mice. Boxes indicate 25th to 75th percentiles; whiskers indicate 5th to 95th percentiles; and the line indicates the median (n=10 per group; 5 male and 5 female). Statistical error analysis was performed by two-way ANOVA with Bonferroni correction, **$p<0.01$, ***$p<0.001$ and ****$p<0.0001$ for the indicated comparisons.

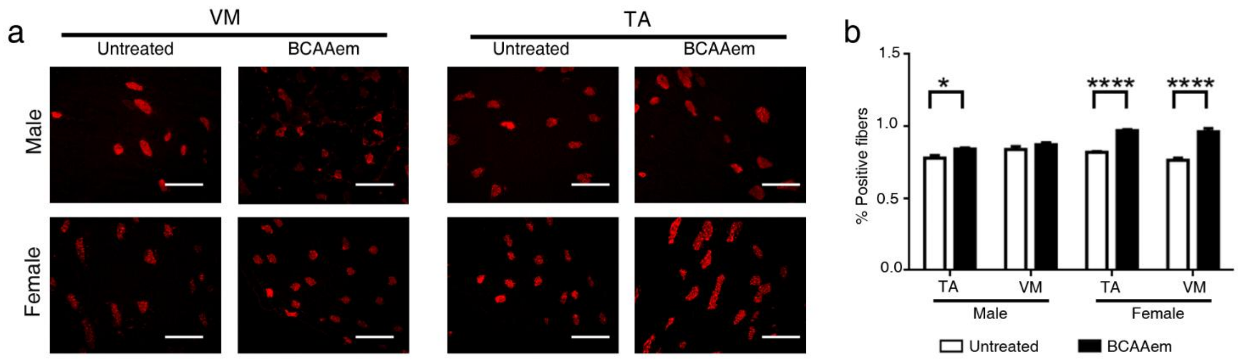

Supplementary Figure 3

Immunofluorescence staining of fast and slow MyHC isoforms from the VM and TA muscle sections of BCAAem-treated and untreated male and female *mdx* mice

Immunofluorescence staining of fast and slow MyHC isoforms from the VM and TA muscle sections of BCAAem-treated and untreated male and female *mdx* mice (**a**) (scale bar, 100 µm). The fluorescence of the MyHC isoforms was quantified with ImageJ software 6.0, and the corresponding histograms are reported as positive fibres per section for male (upper graph) and female (lower graph) samples (**b**). All data are presented as the mean ± s.e.m.; significant differences from the untreated group are indicated *p < 0.05 and ****p<0.0001; two-way ANOVA.

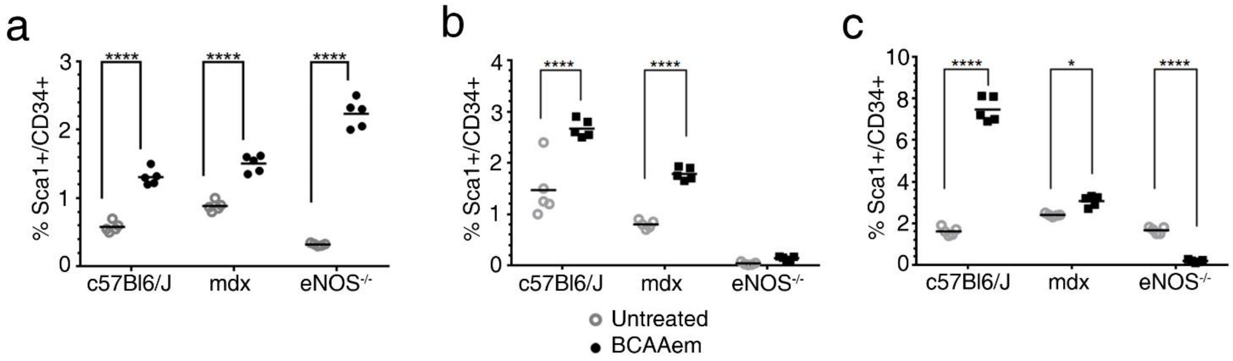

Supplementary Figure 4

BCAAem promotes increased levels of circulating and muscle-derived endothelial progenitors in dystrophic *mdx* mice. Flow cytometric analysis was performed on blood and muscle cells of BCAAem-treated and untreated *C57BL6/J*, *mdx* and *eNOS*$^{-/-}$ mice (n=5 per group). Individual values of SCA1+CD34+ endothelial progenitors (EPs) are reported in the graphs, and the line indicates the mean value in blood (**a**) and in TA (**b**) and VM (**c**) muscle tissues. The increased level of SCA1+CD34+ EPs in the blood of BCAAem-treated mice suggests that EP mobilization was induced by dietary supplementation. Statistical error analysis was performed by two-way ANOVA with Bonferroni correction; *p<0.05 and ****p<0.0001 indicate comparisons that reflect significant differences relative to the untreated group.

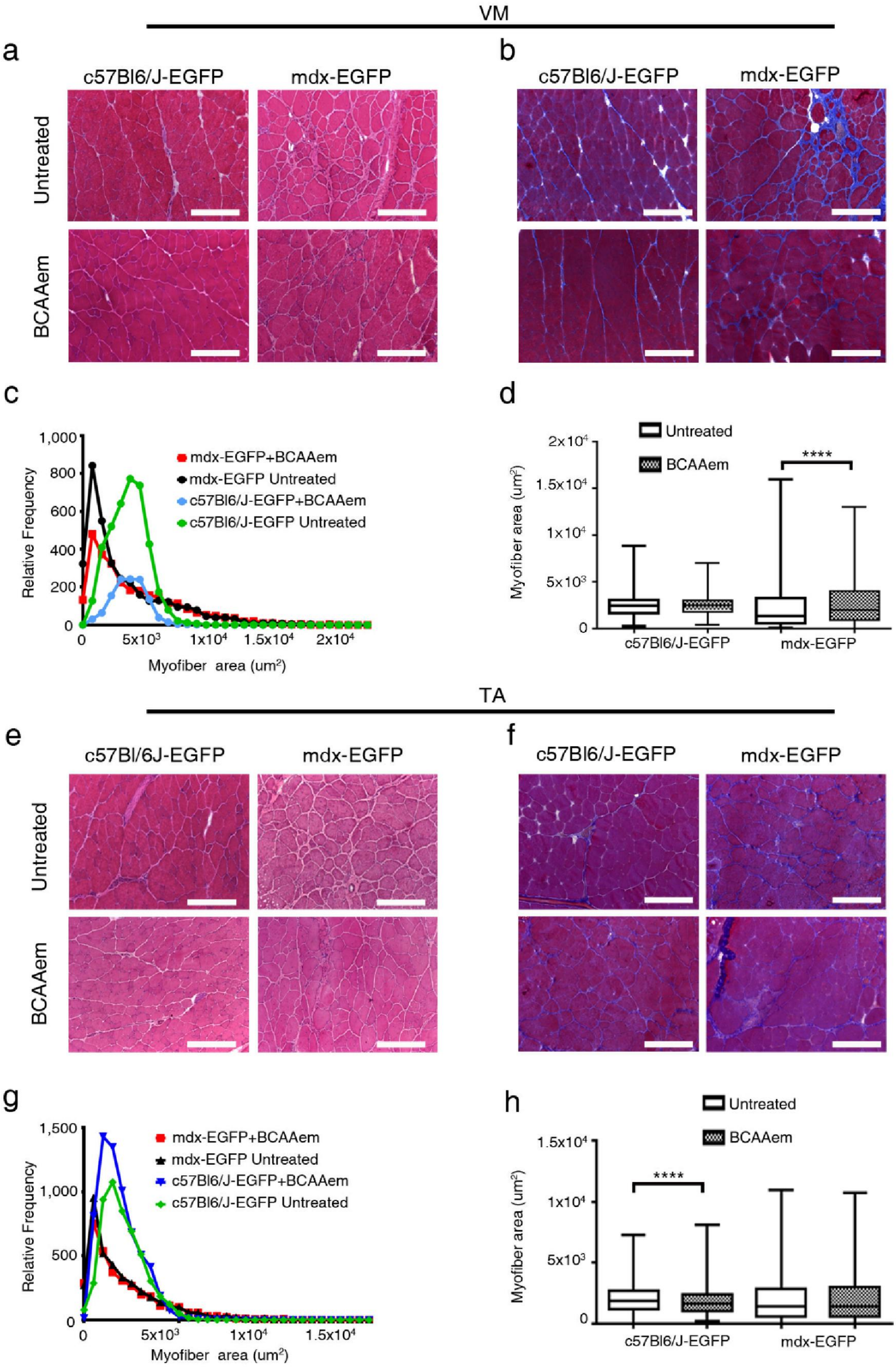
VM
a
c57Bl6/J-EGFP
mdx-EGFP
Untreated
BCAAem
b
c57Bl6/J-EGFP
mdx-EGFP
c
mdx-EGFP+BCAAem
mdx-EGFP Untreated
c57Bl6/J-EGFP+BCAAem
c57Bl6/J-EGFP Untreated
Relative Frequency
Myofiber area (um²)
d
Untreated
BCAAem
****
Myofiber area (um²)
c57Bl6/J-EGFP
mdx-EGFP
TA
e
c57Bl/6J-EGFP
mdx-EGFP
Untreated
BCAAem
f
c57Bl6/J-EGFP
mdx-EGFP
g
mdx-EGFP+BCAAem
mdx-EGFP Untreated
c57Bl6/J-EGFP+BCAAem
c57Bl6/J-EGFP Untreated
Relative Frequency
Myofiber area (um²)
h
Untreated
BCAAem
****
Myofiber area (um²)
c57Bl6/J-EGFP
mdx-EGFP

Supplementary Figure 5

H&E and AM staining of BCAAem-treated and untreated *C57BL6/J-EGFP* and *mdx-EGFP* mice. VM (**a** and **b**) and TA (**e** and **f**) muscle morphology of BCAAem-treated and untreated *C57BL6/J-EGFP* and *mdx-EGFP* mice was evaluated by H&E (**a** and **e**) and AM (**b** and **f**) staining (n=5 for each experimental group). Scale bars, 200 µm. Quantification of the relative frequency of the myofibre cross-sectional area (CSA), expressed as the frequency distribution of the VM (**c**) and TA (**g**) muscles of BCAAem-treated and untreated mice, revealed statistically significant modifications of the CSA in the VMs of dystrophic *mdx-EGFP* mice and TAs of healthy *C57BL6/J-EGFP* mice. Boxes indicate 25th to 75th percentiles; whiskers indicate 5th to 95th percentiles; and the line indicates the median of the VM (**d**) and TA (**h**) muscles of untreated and BCAAem-treated mice (n=10 per group). Statistical error analysis was performed by two-way ANOVA with Bonferroni correction; ****p<0.0001 indicate comparisons that reflect significant differences relative to the untreated group.

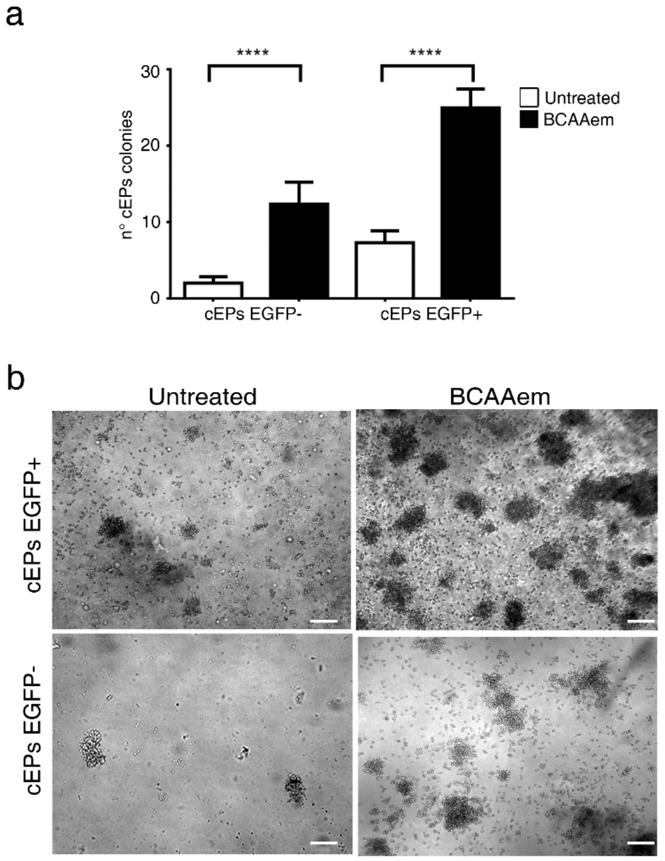

Supplementary Figure 6

BCAAem-treated circulating *EGFP* endothelial progenitors showed increased endothelial colony formation. Colony-forming efficiency of circulating SCA-1+CD34+EGFP± EPs (EGFP± EPs, EGFP+ derived from donor animal and EGFP- derived from recipient animal) isolated from BCAAem-treated and untreated *mdx-EGFP* mice. (**a**) EGFP± EPs isolated from BCAAem-treated *mdx-EGFP* mice had significantly higher colony-forming capacity than the EGFP± EPs from untreated mice. (**b**) Representative images of colonies from the EGFP± EPs from BCAAem-treated and untreated mice and the unsorted populations seeded at 100 or 300 cells/cm^2. Statistical error analysis was performed by one-way ANOVA with Bonferroni correction; ****p<0.0001 indicate comparisons that reflect significant differences relative to the untreated group.

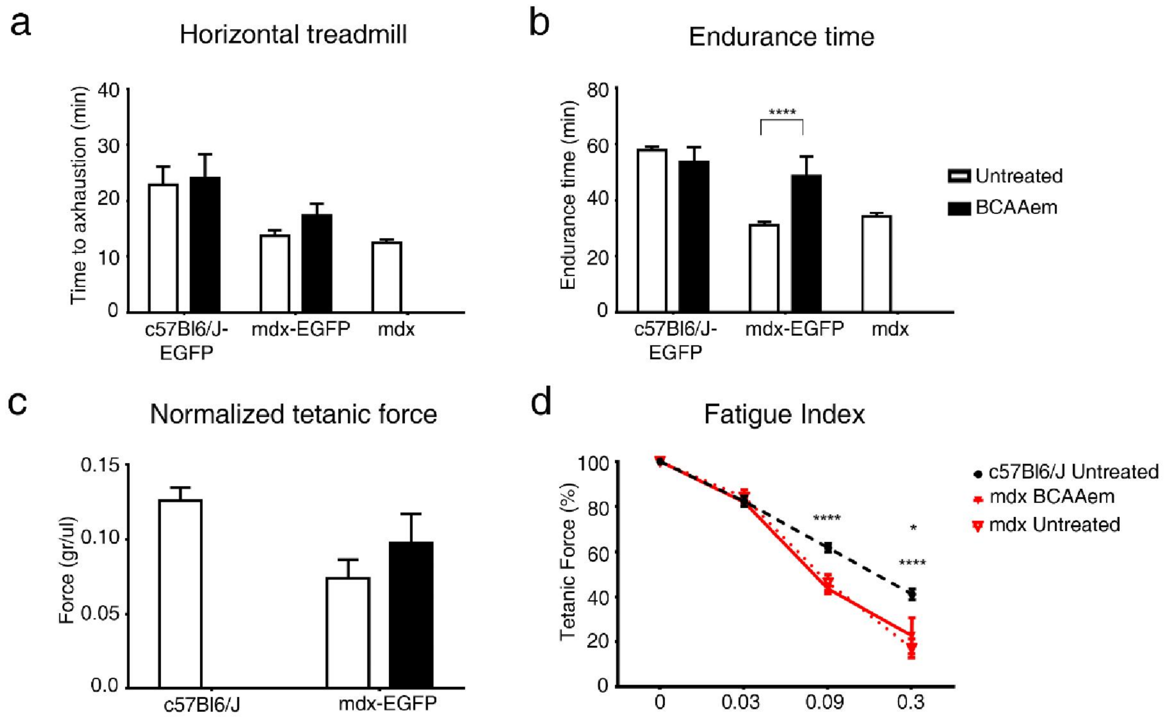

Supplementary Figure 7

BCAAem increases fatigue resistance in *mdx-EGFP* mice. Muscle function of BCAAem-treated and untreated *C57BL6/J-EGFP* and *mdx-EGFP* mice was determined by an incremental treadmill exhaustion test. Exhaustion time is expressed in min (n=5 for each group) (**a**). *In vivo* endurance time is expressed in min (**b**). Muscle function was evaluated in vitro, by measuring the tetanic force (g/µL) of single fibres isolated from TA strips (**c**). Fatigue index, defined as the decrease in tetanic force at different stimulation frequencies (from 0.03 to 0.3 Hz), is compared to the maximal tetanic force (Tf) and expressed in percentage (**d**). **** $p<0.0001$ at 0.09 and 0.3 Hz indicates the comparison between BCAAem-treated or untreated *C57BL6/J-EGFP* and *mdx-EGFP* mice, respectively; * $p<0.05$ at 0.3 Hz indicates the comparison between BCAAem-treated and untreated *mdx-EGFP* mice. All data are presented

as the mean ± s.e.m. Statistical error analysis was performed by two-way ANOVA with Bonferroni correction; ****p<0.0001 indicate comparisons that reflect significant differences relative to the untreated group.

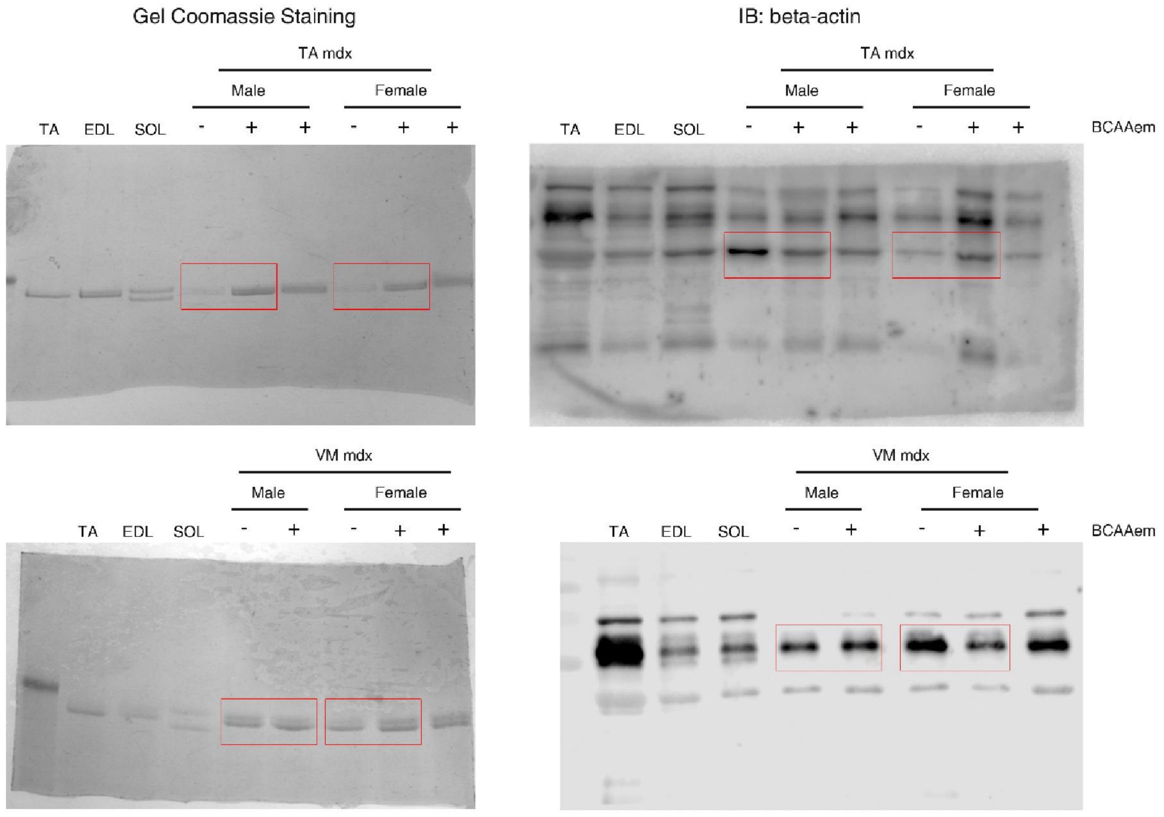

Figure Supplementary 8

Full length gels and blots of Figure 2 a. Red lines show the cropping locations.

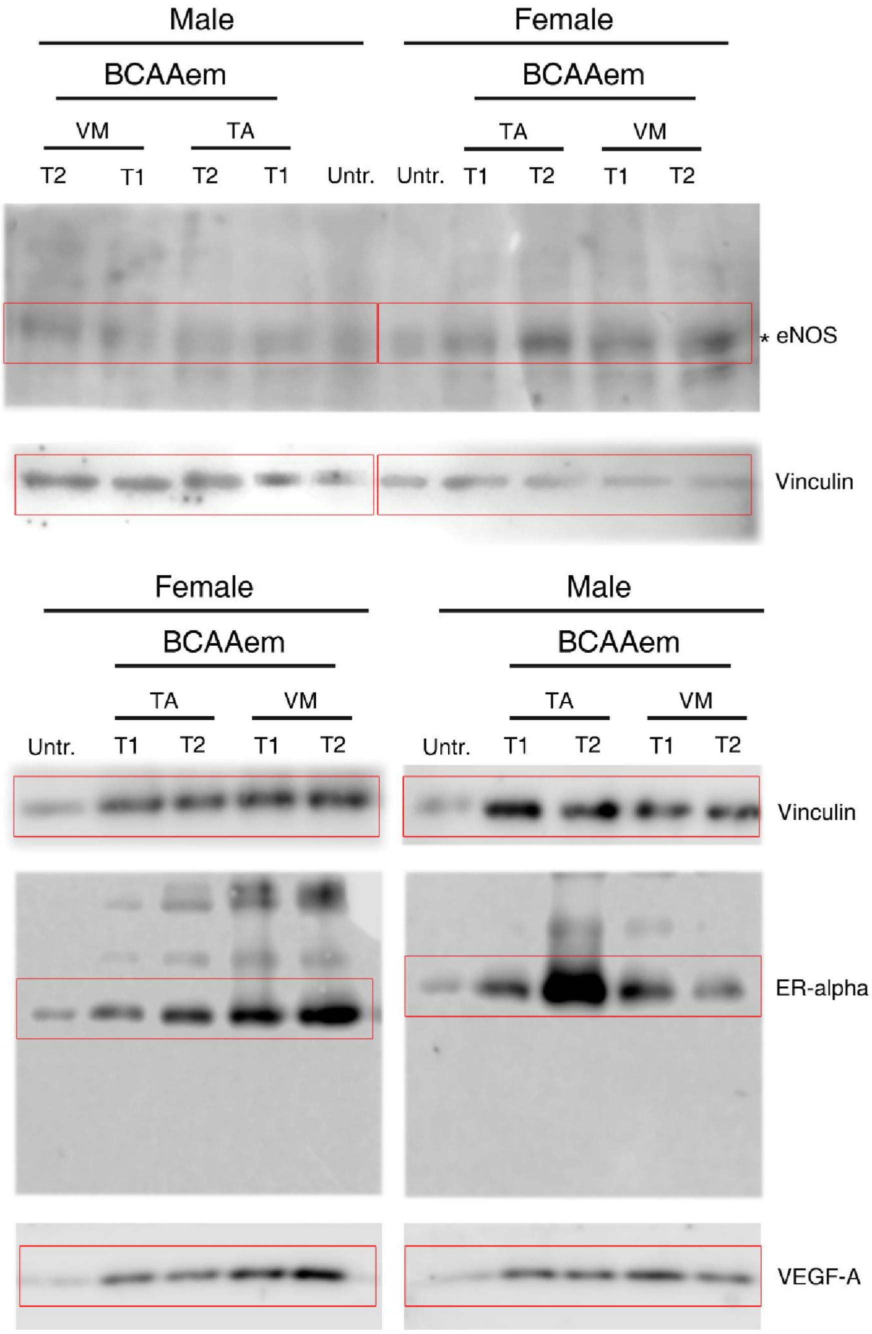

Figure Supplementary 9

Full length blots of Figure 4 a. Red lines show the cropping locations. Brightness was adjusted during processing this gel.

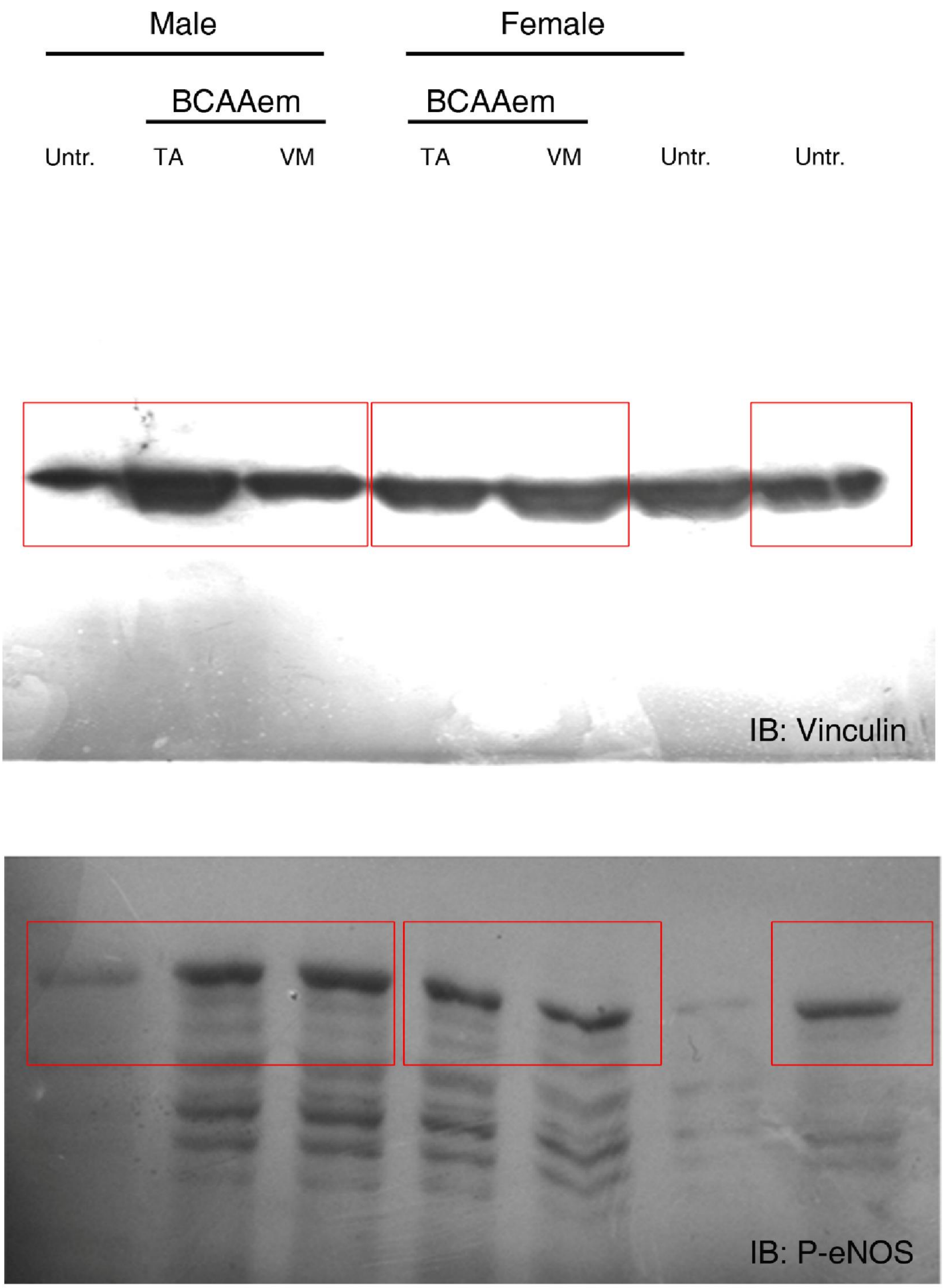

Figure Supplementary 10

Full length blots of Figure 4 c. Red lines show the cropping locations.
